# Interstitial Fluid Shear Stress Induces the Synthetic Phenotype Switching of VSMCs to Release Pro-calcified Extracellular Vesicles via EGFR-MAPK-KLF5 Pathway

**DOI:** 10.7150/ijbs.90725

**Published:** 2024-04-29

**Authors:** Wenbo Gao, Kaiyun Gu, Lunjie Ma, Fan Yang, Li Deng, Yaojia Zhang, Michael Z. Miao, Wenjun Li, Gang Li, Hong Qian, Zhen Zhang, Guixue Wang, Hongchi Yu, Xiaoheng Liu

**Affiliations:** 1 Institute of Biomedical Engineering, West China School of Basic Medical Sciences & Forensic Medicine, Sichuan University, Chengdu 610041, China.; 2 Department of Cardiac Surgery, Children's Hospital, Zhejiang University School of Medicine, National Clinical Research Center for Child Health, Hangzhou 310052, China.; 3 Division of Oral & Craniofacial Health Sciences, Adams School of Dentistry, University of North Carolina at Chapel Hill, NC, 27599, USA.; 4 Department of Genome Sciences, University of Washington, William H. Foege Hall, 3720 15th Ave NE, Seattle 98195, USA.; 5 Department of Cardiovascular Surgery, West China Hospital of Sichuan University, Chengdu 610041, China.; 6 Department of Cardiology, The Third People's Hospital of Chengdu, Affiliated Hospital of Southwest Jiaotong University, Chengdu 610031, China.; 7 Key Laboratory for Biorheological Science and Technology of Ministry of Education, State and Local Joint Engineering Laboratory for Vascular Implants, Bioengineering College of Chongqing University, Chongqing 400030, China.; 8 JinFeng Laboratory, Chongqing 401329, China.

**Keywords:** phenotype switch, interstitial fluid shear stress, extracellular vesicle, KLF5

## Abstract

Phenotypic switching (from contractile to synthetic) of vascular smooth muscle cells (VSMCs) is essential in the progression of atherosclerosis. The damaged endothelium in the atherosclerotic artery exposes VSMCs to increased interstitial fluid shear stress (IFSS). However, the precise mechanisms by which increased IFSS influences VSMCs phenotypic switching are unrevealed. Here, we employed advanced numerical simulations to calculate IFSS values accurately based on parameters acquired from patient samples. We then carefully investigated the phenotypic switching and extracellular vesicles (EVs) secretion of VSMCs under various IFSS conditions. By employing a comprehensive set of approaches, we found that VSMCs exhibited synthetic phenotype upon atherosclerotic IFSS. This synthetic phenotype is the upstream regulator for the enhanced secretion of pro-calcified EVs. Mechanistically, as a mechanotransducer, the epidermal growth factor receptor (EGFR) initiates the flow-based mechanical cues to MAPK signaling pathway, facilitating the nuclear accumulation of the transcription factor krüppel-like factor 5 (KLF5). Furthermore, pharmacological inhibiting either EGFR or MAPK signaling pathway blocks the nuclear accumulation of KLF5 and finally results in the maintenance of contractile VSMCs even under increased IFSS stimulation. Collectively, targeting this signaling pathway holds potential as a novel therapeutic strategy to inhibit VSMCs phenotypic switching and mitigate the progression of atherosclerosis.

## Introduction

Cardiovascular diseases (CVDs) remain the leading cause of death worldwide 1. Atherosclerosis (AS) is a major pathological feature of many CVDs and can lead to myocardial infarction, ischemic cardiomyopathy, stroke, and peripheral arterial disease 2, 3. Vascular calcification, characterized by the ectopic deposition of calcium and phosphate crystals in the blood vessel wall, is a prominent feature of advanced atherosclerotic arteries. During the progression of atherosclerotic lesions, vascular smooth muscle cells (VSMCs) undergo phenotype switching, transition from a contractile phenotype, characterized by markers such as smooth muscle α-actin (α-SMA), smooth muscle protein 22-α (SM22α), calponin, and myosin heavy chain 11, to a synthetic phenotype. This synthetic phenotype, indicated by markers like matrix metalloproteinase-2 (MMP2), and MMP9, enhances the proliferative and migratory capabilities of VSMCs 4, 5. This “contractile to synthetic” phenotype transition is a critical factor in the development of atherosclerosis 6. Current pharmacological treatments primarily target low-density lipoprotein cholesterol, which is associated with major risk factors such as hypercholesterolemia, hypertension, and diabetes 7. However, these therapies have limited direct impact on the VSMCs per se. Percutaneous coronary intervention with a stent has significantly reduced mortality rates in patients with acute coronary syndromes 8, but the development of neo-atherosclerosis, also known as in-stent restenosis, has emerged as a major safety concern due to the increased proliferative VSMCs resulting from phenotypic switching 9. Therefore, directly targeting the phenotypic switching of VSMCs holds therapeutic promise, particularly for patients with CVDs who have relatively normal cholesterol levels, those who experience recurrent CVDs despite lipid-lowering therapy, and those who develop in-stent restenosis after coronary stent implantation.

In healthy vasculature, biomechanical forces play a fundamental role in regulating signaling and gene expression, maintaining vascular homeostasis, and ensuring optimal vascular function 10. Axial stress from longitudinal stretching and circumferential stress generated by cyclic strain induce these forces. Such mechanical stimuli have been found to trigger the release of vasoactive substances, including nitric oxide (NO), through the phosphatidylinositol-3-OH kinase (PI3K)/Akt pathway and Ca^2+^/calmodulin signaling 11. Additionally, these mechanical forces influence the diffusion and modulation of various chemokines and cytokines within VSMCs. In a healthy artery with intact endothelial coverage on the intimal layer, medial VSMCs do not sense changes in wall shear stresses caused by blood flow. Instead, they perceive transmural interstitial flow driven by the transmural pressure gradient 12. However, in the presence of endothelial damage in an atherosclerotic artery, VSMCs are exposed to elevated transmural pressure gradients, leading to increased interstitial fluid shear stress (IFSS) caused by heightened interstitial flow. IFSS has been identified as a regulator of various VSMCs behaviors 10, 12-14. Research has shown that the surface glycocalyx mediates VSMCs contraction and inhibits proliferation upon IFSS stimulation 13, 15. However, accurately measuring the precise value of IFSS experienced by VSMCs remains a challenge, limiting research investigating the contribution of IFSS to VSMCs phenotypic switching. As a result, the molecular mechanotransduction process through which IFSS mediates VSMCs phenotypic switching remains unclear.

Synthetic VSMCs actively participate in intimal calcification through various mechanisms, including apoptosis, osteo/chondrogenic trans-differentiation, and cellular senescence 16-18. Extracellular vehicles (EVs) are membrane-bound microparticles released by cells and are crucial for cell-to-cell communication, transferring molecules like proteins and RNA, which significantly impact physiological and pathological processes. It is well-known that they are key players in diseases such as cancer, cardiovascular diseases, and neurodegenerative disorders, making them potential biomarkers for diagnosis and prognosis 19. Additionally, their therapeutic potential is being explored in drug delivery and regenerative medicine due to their natural cargo-carrying capabilities, biocompatibility, and role in immune system modulation 19. Recent evidence suggests that EVs derived from synthetic VSMCs play a significant role in mediating intimal calcification 20. These EVs are released into the extracellular space and provide nucleation sites for vascular calcification. Conversely, EVs derived from platelet induced VSMCs migration and proliferation 21. Therefore, there likely exists a complex interplay between VSMCs phenotypic switching and EVs secretion, necessitating further research to elucidate the specific mechanisms involved. On the other hand, some literatures suggested that there is a correlation between mechanical stress and EVs biogenesis. For instance, the release of microparticle, a kind of EVs, are inhibited by higher shear in endothelial cells. The loading of cyclic stretch on VSMCs has discrepancies on EVs yield. 18% cyclic stretch (at 1 Hz) increases 2- and 3-fold EVs production in VSMCs 22, while 12% cyclic stretch (at 1 Hz) loading does not show changes in EVs production 23. However, it cannot be refrained from posing the question that what the role of increased IFSS plays in the VSMCs-derived EVs secretion.

In this study, we directly examined human samples from both healthy individuals and patients with atherosclerosis, establishing a direct connection to the disease. Our investigation revealed a clear association between the “contractile to synthetic” phenotype switching of VSMCs and the significant release of EVs in atherosclerotic tissues. To accurately evaluate the impact of increased IFSS, we employed numerical simulations incorporating parameters including fluid viscosity, dynamic viscosity, and porosity, representing different layers of the artery. Subsequent *in vitro* validation confirmed the pivotal role of IFSS in regulating VSMCs phenotypic switching. Notably, there is a substantial increase in EV release following VSMCs phenotypic switching, thus promoting vascular calcification. Mechanistically, the transcriptional factor krüppel-like factor 5 (KLF5) accumulated in the nucleus of VSMCs after exposure to increased IFSS. Blocking KLF5 conversion restored synthetic VSMCs to the contractile state even under elevated IFSS stimulation. Furthermore, our research conclusively establishes the membrane-bound receptor epidermal growth factor receptor (EGFR) and its downstream MAPK signaling pathway as the upstream regulator of KLF5, mediating the transduction of flow-based mechanical cues. This work highlights the central role of EGFR-MAPK-KLF5 signaling in the mechanosensory transduction machinery that drives IFSS-induced phenotypic switching of VSMCs. This knowledge provides valuable insights into potential therapeutic targets for inhibiting VSMCs phenotypic switching in the treatment of atherosclerosis, offering a novel avenue for intervention in this disease.

## Materials and Methods

### Human Artery Samples

Normal artery tissue samples were obtained from heart transplant donors, while atherosclerotic artery tissue samples were collected from heart transplant recipients. The collection process strictly adhered to approved guidelines and obtained informed consent from all participating individuals. The study received ethical approval from the institutional Medical Ethics Committee, ensuring compliance with ethical standards.

### Cell Cultures

Human aortic smooth muscle cells (HASMCs, ScienCell) were cultured in Smooth Muscle Cell medium (SMCM, ScienCell), which consists of 500 mL of basal medium, 10 mL of fetal bovine serum (FBS), 5 mL of smooth muscle cell growth supplement, and 5 mL of penicillin/streptomycin solution. The cells were maintained in a 5% CO_2_ humidified incubator at 37 °C with the medium-replenished every two days. For calcified HASMCs culturing, the osteogenic induction medium (OM) is employed. OM, comprised of high-glucose Dulbecco's Modified Eagle Medium (DMEM), is supplemented with 10% FBS, 1% dual antibiotics, 10 mM β-glycerophosphate, 50 µg/mL ascorbic acid, and 100 nmol/L dexamethasone. Heparin (200 U/mL for 5 days) was used to maintain the contractile phenotype, setting up as the negative control. Additionally, the HASMCs subjected to PDGF-BB (20 ng/mL for 1 day) were used to mimic the synthetic phenotype and regarded as positive control.

### Animals

C57BL/6 mice and apolipoprotein E-deficient (*ApoE^-/-^*) mice were procured from DOSSY Experimental Animal Co., LTD. The animal study was conducted in accordance with the guidelines set by the China Council on Animal Care and was approved by the Medical Ethics Committee of Sichuan University. The C57BL/6 mice were housed in standard cages with a normal diet and unrestricted access to water and maintained under a 12 h light/dark cycle. On the other hand, the atherosclerotic *ApoE^-/-^* mice were fed a high-fat diet for 12 weeks 24. Before the experiments, all mice underwent a minimum of three days of acclimatization. They were housed at a temperature of 20 ± 2°C with 50% ± 10% humidity while being protected from ultraviolet light, noise, and strong odors.

### Calculation of Interstitial Fluid Shear Stress

Blood vessels were collected from both control and atherosclerosis (AS) patients and preserved in 4% paraformaldehyde. Afterward, the vessels were embedded in paraffin, cross-sectioned, and subjected to HE staining. The staining results were used to measure the thickness and porosity of the inner, middle, and outer layers of the vessels. Additionally, an idealized computational model of blood vessels has been established. The internal diameter of the vessel was set at 19 mm 25, with a length of 100 mm^26^, to ensure adequate fluid flow. In this study, the structured grid is used to divide the model, and the boundary layer is added to the fluid-solid interface, and the final grid number is 1.43×10^6^. Computational fluid dynamics (CFD) is used to calculate the steady state, and CFD simulation is carried out by using commercial software Ansys Fluent. The incompressible N-S equation is solved by SIMPLE method, and the calculation is considered to be convergent when the residual is less than 10^-5^ and the difference between the outlet and inlet mass flows is less than 0.01% of the outlet mass flow. The simulation calculation uses steady-state calculation, so the time step is not involved.

The N-S equation is as follows:


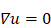

(1)


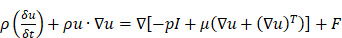

(2)

Where the blood velocity (u), pressure (p), dynamic viscosity (μ), blood density (ρ), and volume force field (F) play crucial roles in understanding the mechanics of blood flow. The blood density was determined as 1. 05 ×10^3^ kg/m^3^, and the viscosity was 0.0035 Pa∙s. The interstitial flow had a density of 1.0 ×10^3^ kg/m^3^ and a viscosity of 0.0035 Pa∙s 27. The vessel wall was modeled as a porous media. The inlet boundary conditions were defined by a lumen blood flow velocity of 5 L/min and an interstitial flow velocity ranging from 0.1-1.0 μm/s 28. The outlet boundary conditions were set at an intraluminal pressure of 70 mmHg 29. Physiological/pathological parameters and boundary conditions used in the numerical model are listed in Table 1. The thickness and porosity of the vascular model are all measured according to the HE staining of human vascular samples, which are detected by FIJI ImageJ (Version 1.53).

In the CFD calculation, the model is simplified to a certain extent, and the specific value is the average value of the wall shear stress (*WSS*) at the interface between the endothelial cell layer and the smooth muscle cell layer. The *WSS* is the projection of the traction vector in the direction tangent to the wall 30.



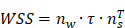



where 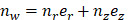
is the unit normal vector to the surface of interest. 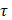
is the Cauchy stress tensor
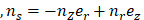
is the unit vector tangent to the wall surface. The surface traction vector, 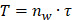 is the inner product of the Cauchy stress tensor and the unit normal vector to the surface of interest.

### IFSS Loading Condition

The IFSS loading system utilized in this study consisted of a parallel plate flow chamber and a peristaltic pump system (Masterflex model 7518-10, Cole-Parmer Instrument Company). HASMCs were exposed to IFSS in this loading system. The calculation of the loading shear stress was based on a previously described formula 31:



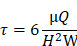



In the formula, τ represents the IFSS applied to the cells; μ (0.83 mPa∙s) represents the viscosity of the circulating buffer; *Q* represents the velocity of the flow; H (0.4 mm) represents the height of the chamber; and W (95 mm) represents the width of the chamber. According to the above formula, when τ is set at 0.5 or 3 dyn/cm^2^, the corresponding *Q* value is 9 or 55 mL/min, respectively. During the 8 h IFSS loading process, the loading system was placed in a 37°C, 5% CO_2_ incubator. To investigate the role of EGFR and MAPK signaling pathway, HASMCs were pretreated with the EGFR inhibitor gefitinib (1 μM) for 48 h and MEK inhibitor PD9805 (10 μM) for 24 h before IFSS loading.

### Statistical Analysis

To minimize potential environmental bias or unintentional error, quantitative experiments were performed at least three times. SigmaPlot software (Systat, Erkrath) was used for statistical analyses. Depending on the results of the Shapiro-Wilk test for normal distribution, either Student's t-test or the non-parametric Mann-Whitney U rank sum test was used for statistical analysis. The error bars in the bar graphs represent the standard errors of the mean (SEM). In the figures and legends, the corresponding *P* values are indicated by symbols such as ^*^ *P* < 0.05,^ **^ *P* < 0.01, ^***^ *P* < 0.001, and n.s. for no significance. GraphPad Prism 8.0 software (La Jolla) was used to generate the graphs.

## Results

### The Phenotypic Switching of VSMCs Is Accompanied by an Increase of EVs in the Atherosclerotic Artery

**Figure 1:**
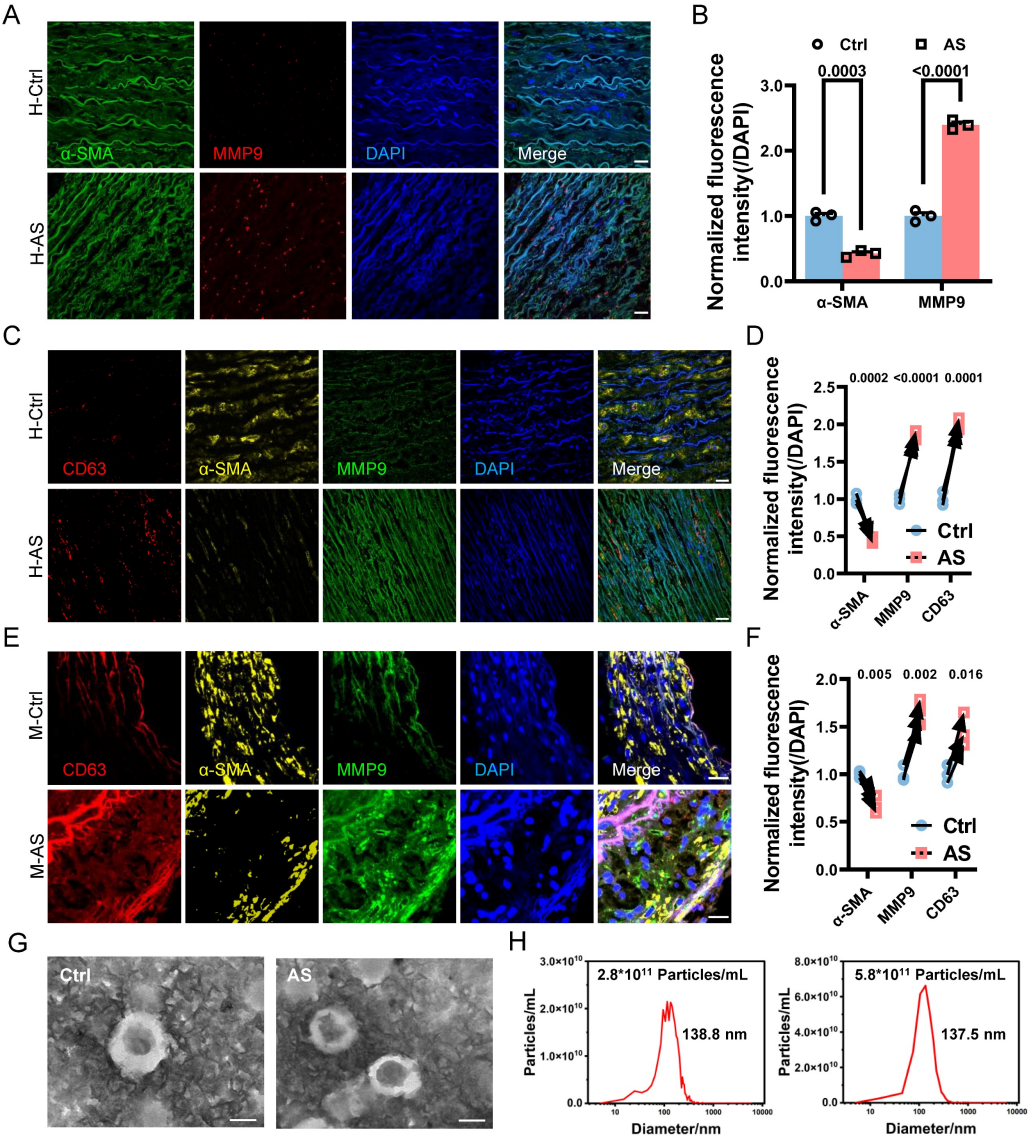
** Phenotypic switching of VSMCs is associated with increased EVs in atherosclerotic plaque.** (**A, B**) Immunostaining and the normalized intensities show the expression change of phenotype switching indicators in VSMCs from the atherosclerosis plaque, α-SMA (the contractile marker, red), MMP9 (synthetic marker, green), and DAPI (nucleus, blue). Scale bar, 20 μm, n=3. (**C**) Immunostaining exhibits the location and expression of VSMCs phenotype (α-SMA, yellow, MMP9, green) and extracellular vesicles markers (CD63, red) in human atherosclerotic arteries. Nuclei were counterstained with DAPI (blue). Scale bar, 20 μm, n=3. (**D**) Correlation analysis of VSMCs phenotypic switching markers and EVs markers. (**E, F**) The expression and correlation analysis of VSMCs phenotype and extracellular vesicles markers in the mouse atherosclerotic artery (α-SMA, yellow, MMP9, green, CD63, red). Nuclei were counterstained with DAPI (blue). Scale bar, 20 μm, n=3. (**G**) The released EVs from the representative normal and atherosclerotic mouse artery tissue are harvested and identified by the TEM analysis. Scale bar, 100 μm, n=6. (**H**) NTA analysis is used to determine the particle size and number of the EVs harvested in (**G**). Data are presented as mean ± SEM, and statistical analysis was performed by a two-tailed unpaired t-test.

To begin, we conducted a comprehensive analysis to confirm the occurrence of phenotypic switching in VSMCs within human atherosclerotic artery tissue. Utilizing confocal imaging, we observed a notable increase in the expression of synthetic marker matrix metalloproteinase-9 (MMP9), accompanied by a decline in the contractile marker alpha-smooth muscle actin (α-SMA) in the atherosclerotic artery tissue (**Figure 1A** and** 1B**). We further validated this relationship in an *ApoE^-/-^* mouse atherosclerotic model, which is characterized by abundant lipid deposition in the different artery segments (**Figure S1A** and** S1B**). Consistently, phenotypic switching of VSMCs was observed in the media layer leading to a narrowed artery lumen (**Figure S1C-G**). The artery consists of layers including the intima, media, and adventitia, each populated with diverse cells and extracellular matrix proteins, notably elastin and collagens in the media layer, the site of VSMCs 32. The blue, long, wavy structures observed in the media layer in **Figures 1A** and** 1C** likely result from the nonspecific staining of the elastic lamina.

Given the growing evidence implicating EVs in the development and progression of atherosclerosis 33, we further evaluated the secretion of EVs in the atherosclerotic artery. Comparative analysis between normal human arteries and atherosclerotic arteries revealed a significant increase in the colocalization of VSMCs phenotypic switching and the EVs marker CD63 in the atherosclerotic artery (**Figure 1C** and** 1D**). Similarly, phenotypic switching VSMCs in the atherosclerotic mouse aortic arch also exhibited a higher expression level in CD63 (**Figure 1E** and** 1F**). This positive correlation between CD63 expression and VSMCs phenotypic switching was also observed in other artery segments of atherosclerotic mice (**Figure S2 A-D**).

To further verify the association between VSMCs phenotypic switching and increased EV release, we performed transmission electron microscopy (TEM) and nanosight tracking analysis (NTA) to characterize and accurately quantify EVs in the atherosclerotic artery. The isolated EVs exhibited the typical double-layered cup-shaped structure (**Figure 1G**) and size distribution (**Figure 1H**) in both normal and atherosclerotic mouse arteries. However, the EVs amount in atherosclerotic mouse artery (5.8×10^11^ particles/mL) was significantly higher than that in normal mouse artery (2.8×10^11^ particles/mL), with a ratio approximately reaching 2.1. These findings strongly suggest that VSMCs undergo phenotypic switching in atherosclerosis, accompanied by an increased release of EVs.

### Increased Interstitial Fluid Shear Stress Drives VSMCs Phenotypic Switching

**Figure 2:**
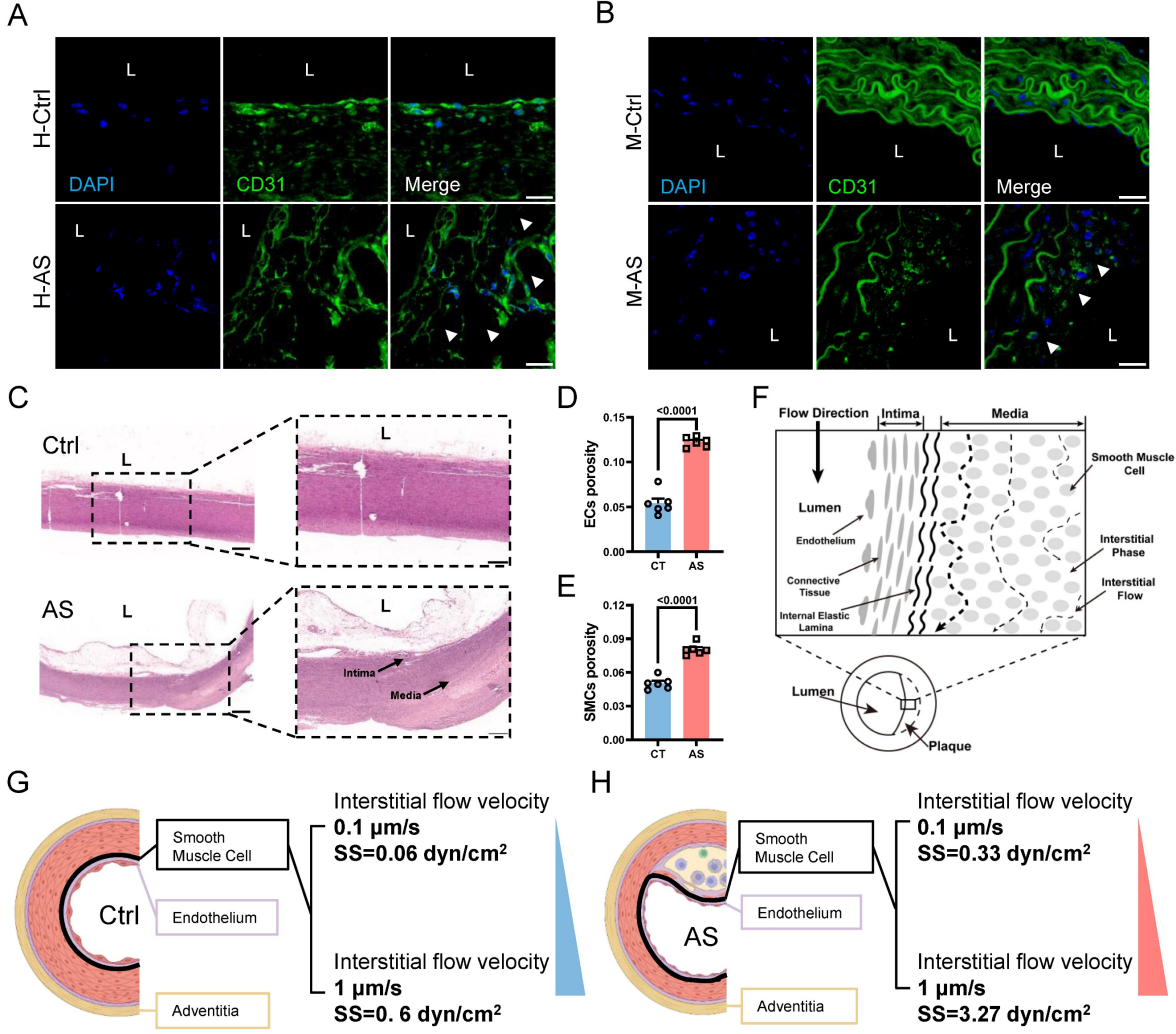
** The remodeling of atherosclerotic artery leads to the increased interstitial flow.** (**A, B**) Immunostaining illustrates the remodeling of the intimal and medial layer in the normal and atherosclerotic artery tissues from the representative human (**A**) and mice (**B**), respectively. White arrows indicate the impaired endothelial layer. CD31 (endothelial cells indicator, green), nucleus (DAPI, blue), L denotes the vascular lumen. Scale bar, 20 μm, n=3. (**C**) HE staining of normal and atherosclerotic arteries from humans reveals the remodeling of the intima and media layers. Scale bar, 500 μm, n=3. (**D, E**) Quantification of porosity in the intimal endothelial cells and the medial VSMCs, n=6. Unpaired t-tests were used to compare variables with normal distribution and homogeneity of variance. All data are presented as mean ± SEM. (**F**) A schematic diagram of aortic remodeling induced the change of interstitial flow. All three dashed lines represent interstitial flow, with the interstitial flow near the intima layer being the highest (the first black dashed line on the left). (**G**) Numerical calculation of IFSS subjected to VSMCs in the media layer near the intima layer (thick black line) is based on the physiological parameters of the normal human artery. The inlet flow speed ranges from 0.1 μm/s to 1 μm/s and the magnitude of IFSS ranges from 0.06 to 0.6 dyn/cm^2^. SS: shear stress. (**H**) Numerical calculation of IFSS subjected to VSMCs in the media layer near the intima layer (thick black line) according to the pathological parameters of the atherosclerotic human artery. The inlet flow speed ranges from 0.1 μm/s to 1 μm/s and the magnitude of IFSS ranged from 0.33 to 3.27 dyn/cm^2^. SS: shear stress.

The phenotypic switching of VSMCs is known to be responsive to various pathological stimuli, including pro-inflammatory cytokines and biomechanical factors 4. In the context of atherosclerosis progression, the damaged endothelium leads to the modulation of various VSMCs behaviors through increased IFSS 10, 12, 13. However, it remains unclear whether increased IFSS directly regulates phenotypic switching. Furthermore, the accurate measurement of IFSS *in vivo* poses a challenge, leading to a debate regarding its precise value when VSMCs are exposed to it within the atherosclerotic artery. To address these issues, we performed immunofluorescent staining to detect the endothelial layer remodeling and vascular morphology changes in the atherosclerotic artery. Both human and mouse samples of atherosclerotic arteries exhibited irregular endothelial cell arrangement, a loose structure, and the formation of numerous pores (indicated by white arrows) compared to normal arteries (**Figure 2A** and** 2B**). This observation was further confirmed by the HE staining of artery tissue (**Figure 2C**). Quantification of artery porosity based on histological staining revealed an increase from 5.4% to 12.2% in the intimal layer (**Figure 2D**), and from 5.0% to 8% in the medial layer (**Figure 2E**) in atherosclerotic arteries. Although the internal elastic lamina prevented the blood flow from seeping into the medial layer 34, the increased permeability of endothelium and enlarged porosity of the medial layer resulted in an elevated interstitial flow near the intima layer with a gradually reduced tendency (**Figure 2F**). To determine the precise magnitude of IFSS, we performed numerical calculations of interstitial flow near the intima layer, which is highlighted by dark circle in **Figure 2G** and** 2H** (left panel), based on the physiological and atherosclerotic parameters and boundary conditions (detailed information in** Table 1**). Interstitial fluid velocity was set within the range of 0.1 μm/s to 1 μm/s^35^, ^36^. Consequently, we determined that the precise magnitude of IFSS in normal arteries ranged from 0.06 to 0.6 dyn/cm^2^, while in atherosclerotic arteries, it ranged from 0.33 to 3.27 dyn/cm^2^ (**Figure 2G** and **2H**, right panel).

To accurately select the most representative IFSS value regulating phenotypic switching of VSMCs, we utilized the minimum, average, and maximum values derived from our calculations for experimental validation. In addition, heparin was used to maintain the contractile phenotype of VSMCs setting as the negative control group. In contrast, the synthetic VSMCs induced by PDGF-BB were regarded as the positive control group 20, 37. Immunofluorescence staining revealed that VSMCs do not acquire synthetic phenotype under physiological IFSS (≤ 0.5 dyn/cm^2^) condition. In contrast, VSMCs exposure to pathological IFSS (≥1.5 dyn/cm^2^) exhibit a progressive decrease in contraction phenotype markers α-SMA and SM22α and a concomitant increase in synthetic phenotype markers MMP9 and MMP2 (**Figure S3A** and** S3B**). It was supported by the data from western blot assay. Compared with VSMCs upon heparin treatment, the VSMCs subjected to IFSS ≤ 0.5 dyn/cm^2^ do not show significantly different expression of contractile markers (**Figure S3C-E**). Conversely, VSMCs with 3 dyn/cm^2^ IFSS exposure show the most obviously decreased expression in contractile markers (α-SMA and SM22α) and the most remarkably increased expression in synthetic marker (MMP-2). It was consistent with the expression of phenotypic markers in VSMCs cultured in PDGF-BB (**Figure S3C** and** S3F**). Furthermore, the qRT-PCR assay confirmed the expression change of phenotypic markers at gene level (**Figure S3G-K**). Collectively, VSMCs with 0.5 dyn/cm^2^ IFSS stimulation show the most pronounced characterization of contractile phenotype, while VSMCs subjected to 3 dyn/cm^2^ IFSS have the most likely tendency of synthetic marker with that in VSMCs cultured with PDGF-BB. Therefore, we used 0.5 dyn/cm^2^ IFSS as the physiological group and 3 dyn/cm^2^ IFSS as the pathological group.

**Figure 3:**
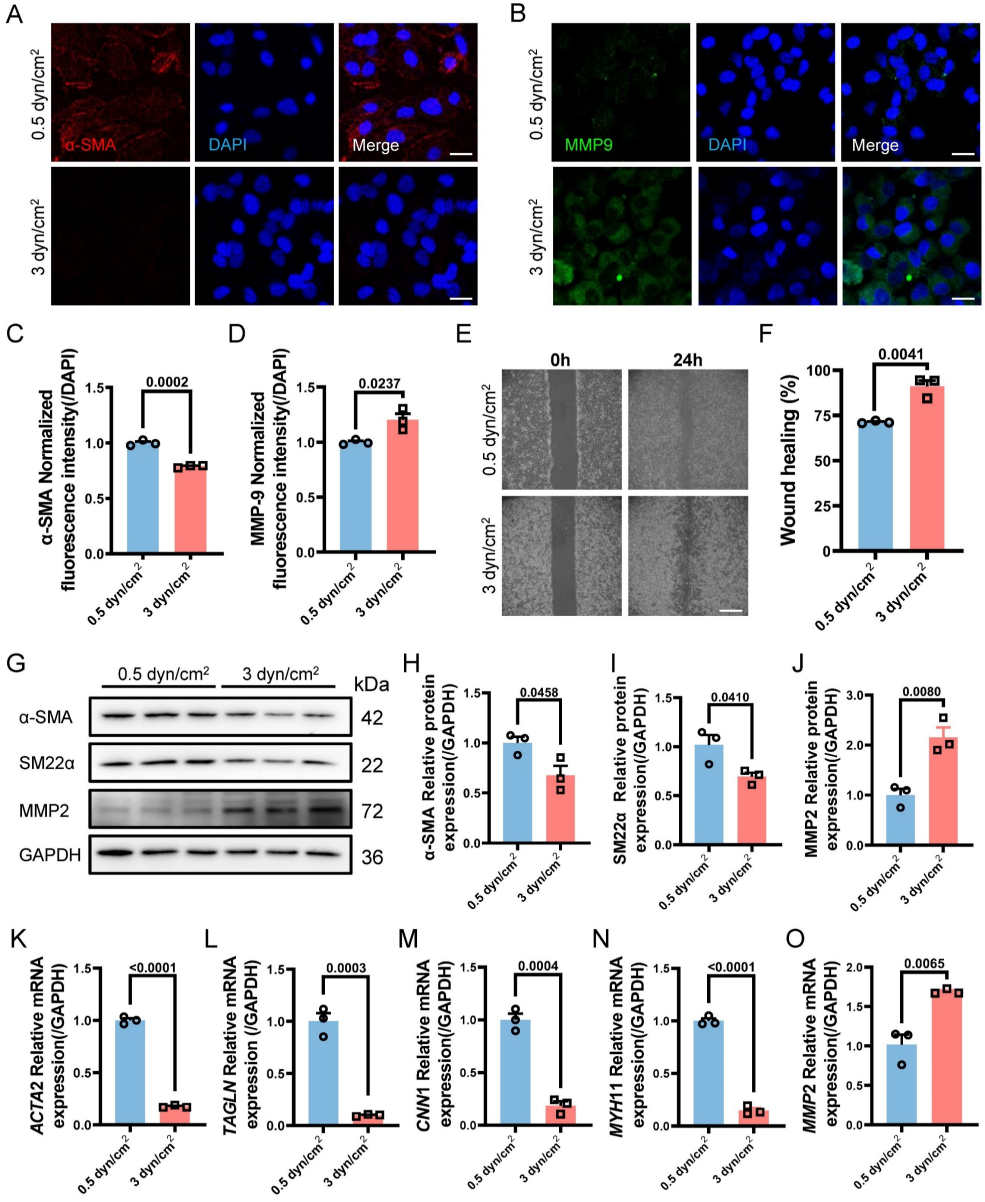
** Increased interstitial flow promotes the phenotype switching of VSMCs.** (**A, B**) Immunostaining indicates the expression changes of phenotype markers (**A**, α-SMA, red), (**B**, MMP9, green) in HASMCs upon increased IFSS stimulation *in vitro*. The nucleus is counterstained with DAPI (blue). Scale bar, 20 μm, n=3. (**C, D**) Normalized fluorescence intensity of α-SMA and MMP9 were quantified in **Figure 3A** and **3B** respectively. (**E, F**) Wound healing assay verified the increased mobility of HASMCs after phenotypic switching upon IFSS stimulation. Scale bar, 200 μm, n=3. (**G-J**) Western blot and quantitative analyses of phenotypic switching indicators in HASMCs exposed to increased IFSS. (**K-O**) mRNA levels of contractile markers (*ACTA*2, *TAGLN*, *CNN*1, and* MYH*11) and synthetic marker (*MMP*2) in HASMCs after IFSS stimulation. The relative abundances of transcripts were quantified and normalized to *Gapdh*, n=3. Data are presented as mean ± SEM, and statistical analysis is performed by a two-tailed unpaired t-test.

Next, we aimed to further verify whether increased IFSS in the medial layer of the atherosclerotic artery is the main driver for VSMCs phenotypic switching *in vitro*. When exposed to increased IFSS stimulation, VSMCs exhibited reduced expression of the contractile marker α-SMA and increased expression of the synthetic marker MMP9 (**Figure 3A**-**D**), indicating that the increased IFSS promoted the phenotypic switching of VSMCs. The elevated motility of VSMCs further confirmed the occurrence of phenotypic switching in response to increased IFSS (**Figure 3E** and** 3F**). Moreover, through western blot and qRT-PCR assays, we found that the contractile phenotype markers α-SMA and SM22α were significantly reduced under 3 dyn/cm^2^ IFSS stimulation, whereas the synthetic phenotype marker MMP2 was significantly upregulated (**Figure 3G-O**). In summary, we utilized numerical simulations to calculate the values of IFSS and *in vitro* validation revealed that 0.5 dyn/cm^2^ and 3 dyn/cm^2^ can simulate the IFSS under physiological and pathological conditions for subsequent experiments.

### VSMCs Phenotypic Switching Serves as the Upstream Regulator of EVs Secreting

After determining the specific magnitudes of IFSS *in vitro*, we attempted to observe whether mechanical loading on VSMCs leads to the release of extracellular vesicles. NTA assay showed that the number of EVs from VSMCs subjected to the increased IFSS reached 4.2 times higher than that from VSMCs exposed to normal IFSS (**Figure S4A** and** S4B**). We assessed the EVs markers CD63 and TSG101 through western blot (**Figure S4C**), and also measured the concentration of EVs (**Figure S4D** and** S4E**), observing a significant increase in the release of EVs after IFSS loading. Besides, we detected the expression of CD63 through immunostaining and qRT-PCR, both of which demonstrated higher expression of CD63 (**Figure S4F**-**H**). Rab27a, a pivotal contributor to EVs biogenesis and secretion, was generally selected to evaluate the release of EVs 38. It has been shown that silencing Rab27a inhibited the biogenesis of EVs 39. In line with the previous research, Rab27a exhibited a significant increase in VSMCs after increased IFSS stimulation (**Figure S4I**-**K**). These results drew our attention, the elevated IFSS could also induce the releasing of EVs-derived from VSMCs.

**Figure 4:**
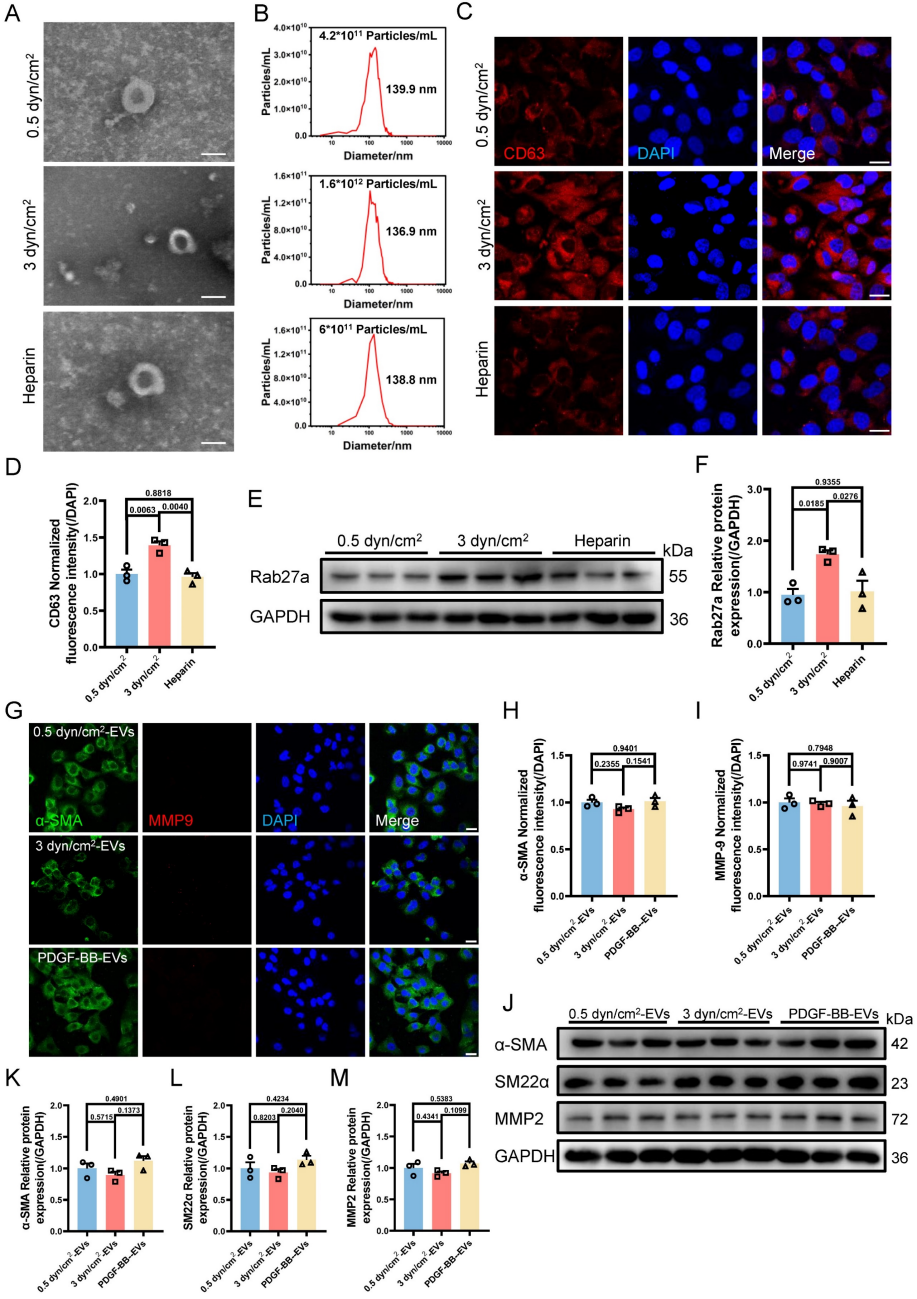
** The increased IFSS-induced phenotypic switching of VSMCs is the upstream factor facilitating the EVs release.** (**A**) TEM analysis is used to identify the HASMCs-derived EVs after increased IFSS stimulation. Heparin is used to maintain the contractile phenotype of HASMCs. Scale bar, 100 μm. (**B**) NTA analysis measured the size and particle number from HASMCs subjected to the indicated stimulation. (**C, D**) Immunostaining and normalized fluorescence intensity analyses reveal the expression of CD63 in HASMCs under the indicated stimulation. CD63, red. DAPI was used to stain nuclei (blue). Scale bar, 20 μm, n=3. (**E, F**) Immunoblotting and quantitative analysis of Rab27a in HASMCs after indicated stimulation, GAPDH is used as a loading control, n=3. (**G-I**) Immunostaining and normalized fluorescence intensity analysis illustrate the phenotypic switching of HASMCs after increased IFSS stimulation. PDGF-BB is co-cultured with HASMCs 8 h and induces the synthetic phenotype of HASMCs. α-SMA (green), MMP9 (red). DAPI was used to stain nuclei (blue). Scale bar, 20 μm, n=3. (**J-M**) Immunoblotting and quantitative analysis of phenotypic switching markers in HASMCs with exposure to HASMCs-derived EVs upon the different IFSS stimulation. GAPDH is used as a loading control, n=3. Data are shown as mean ± SEM, and statistical analysis was performed by one-way analysis of variance followed by the Tukey test.

VSMCs undergo phenotypic switching and secrete EVs in response to increased IFSS in the atherosclerotic artery. The relationship between VSMCs phenotypic switching and EVs secretion remains unclear, as both factors have been shown to influence each other's functions. Low-density lipoprotein (LDL) particles have been shown to promote EVs release from VSMCs into the adjacent tissue 40. Conversely, EVs also influence VSMCs phenotypic switching, thereby contributing to abnormal VSMCs proliferation 41. Subsequently, we sought to unravel the upstream regulator between the two under increased IFSS conditions. Firstly, heparin was used to maintain the contractile phenotype of VSMCs. This treatment group was regarded as the negative control group to compare the physiological simulating efficiency of 0.5 dyn/cm^2^ for keeping VSMCs quiescence. Distinctly, the elevated amount of EVs from VSMCs induced by increased IFSS was observed (**Figure 4A-F**), while 0.5 dyn/cm^2^ and heparin groups do not show the increased EVs. It suggested that the contractile phenotype of VSMCs suppresses the release of EVs from VSMCs.

On the other hand, we established a synthetic phenotype model of VSMCs by using PDGF-BB 37 and extracted EVs from both the PDGF-BB or increased IFSS-induced synthetic VSMCs. Co-culturing VSMCs with EVs derived from either the PDGF-BB-induced or the increased IFSS-induced synthetic VSMCs did not induce phenotypic switching of the recipient cells (**Figure 4G-I**). Furthermore, the western blot analysis revealed that the expression of synthetic phenotype markers marker MMP2 and contractile markers (α-SMA, SM22α) did not show a significant change in VSMCs exposed to EVs derived from synthetic VSMCs (**Figure 4J-M**). These results suggest that EVs-derived from the synthetic VSMCs may be incapable of inducing VSMCs phenotypic switching. Collectively, VSMCs phenotypic switching acts on the upstream regulator of EVs secreting.

**Figure 5:**
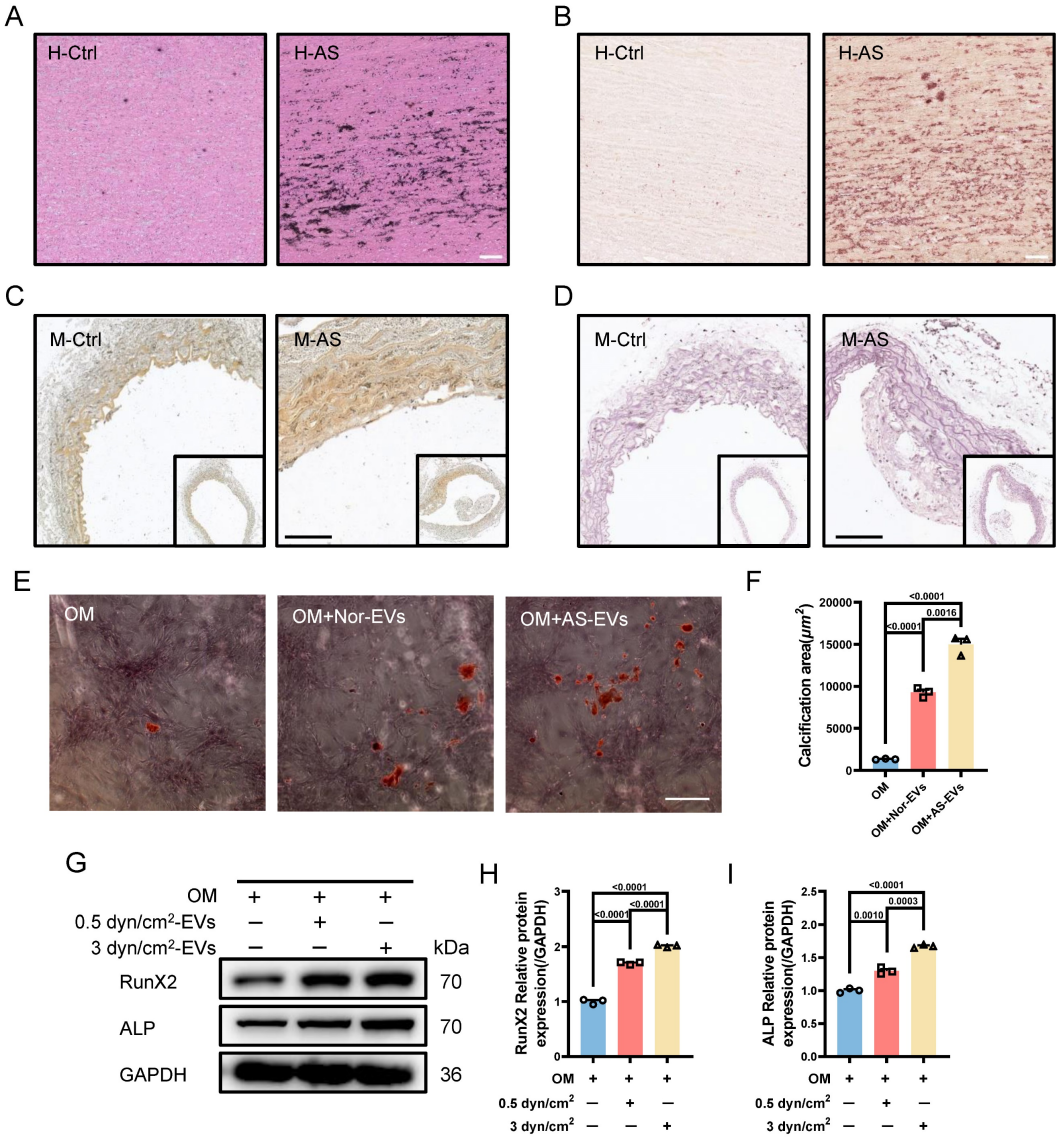
** Synthetic VSMCs-derived EVs promote the calcification of artery.** (**A**) Von Kossa staining shows calcium deposition in human atherosclerotic artery tissue. Scale bar, 100 μm. (**B**) Alizarin red S staining is performed to confirm the calcium deposition in the atherosclerotic artery tissue from human. Scale bar, 100 μm. (**C, D**), Von Kossa and Alizarin red S staining reveal the calcium deposition in the atherosclerotic artery tissue from mouse. Scale bar, 200 μm. (**E, F**) Alizarin red S staining of primary VSMCs isolated from a normal mouse artery subjected to the EVs, which is released from the primary VSMCs isolated from the atherosclerotic mouse artery. Red plaques indicate the calcification nodules. Scale bar, 20 μm. (**G-I**) Immunoblotting and quantitative analysis of calcification marker (RunX2, ALP) in HASMCs co-cultured with the EVs derived from HASMCs subjected to increased IFSS. OM: osteogenic induction medium. GAPDH is used as a loading control, n=3. Data are shown as mean ± SEM. Statistical analysis was performed by one-way analysis of variance followed by the Tukey test.

Based on the above results, another interesting question arises: what is the function of EVs-derived from the synthetic VSMCs induced by increased IFSS? Our subsequent objective was to assess the impact of EVs-derived form the synthetic VSMCs under increased IFSS in the development of atherosclerosis. Vascular calcification is a common feature observed in different stages of the atherosclerotic plaque 42. Consistently, we observed significant calcified nodes in human atherosclerotic artery tissue (**Figure 5A** and** 5B**), aortic arch segments (**Figure 5C** and** 5D**) and other artery segments of atherosclerotic mice (**Figure S5A-F**). The co-localization of EVs with synthetic VSMCs in atherosclerotic arteries suggests that the EVs may play a potential promoter of vascular calcification. To investigate this hypothesis, we isolated EVs from primary VSMCs of the atherosclerotic mouse artery and co-cultured with the primary VSMCs from the normal mouse artery. The results exhibited an increase in calcified nodules following exposure to these EVs, as observed through alizarin red staining (**Figure 5E** and** 5F**). Additionally, *in vitro*, the EVs derived from VSMCs under increased IFSS accelerate the higher expression of calcified markers (RunX2 and ALP) in normal VSMCs (**Figure 5G-I**), suggesting the pro-calcification role of these EVs. These results collectively demonstrated that the EVs derived from synthetic VSMCs do not induce phenotypic switching of recipient VSMCs but do initiate the calcification of VSMCs. Given that increased IFSS triggers phenotypic switching of VSMCs, we hypothesize that increased IFSS serves as the initial factor in vascular calcification during the progress of atherosclerosis.

### Transcription Factor KLF5 is the Executor for VSMCs Phenotypic Switching

**Figure 6:**
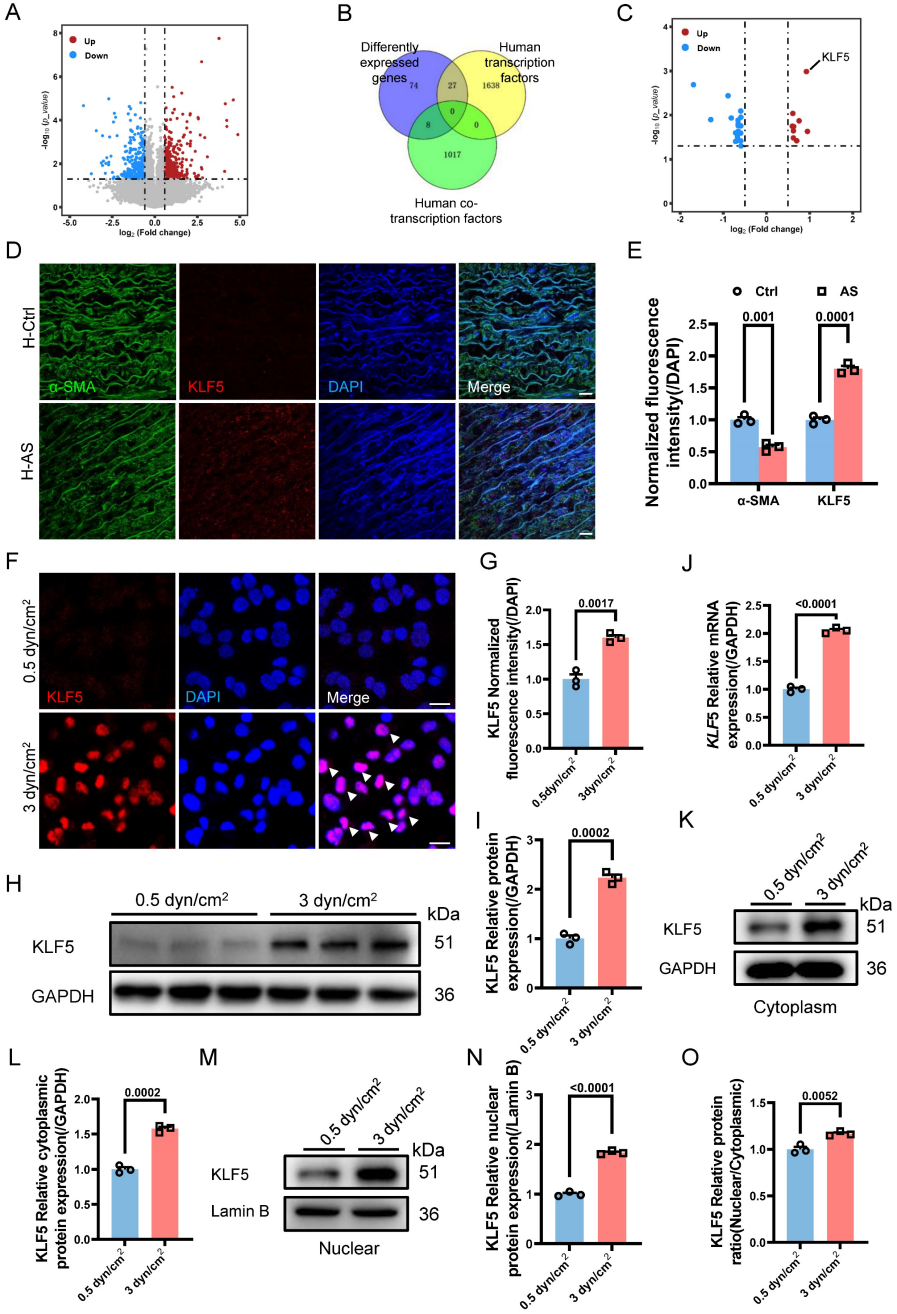
** KLF5 drives IFSS-induced phenotypic switching of VSMCs.** (**A**) Volcano plot of differently expressed genes in HASMCs after subjecting to increased IFSS. Compared to the 0.5 dyn/cm^2^ group, 393 genes were upregulated, 385 genes were downregulated, and 28,545 genes showed no significant difference in the 3 dyn/cm^2^ group. (**B**) Venn analysis of the differently expressed genes with co-transcriptional and transcriptional factors. (**C**) The highest expression level of KLF5 in the intersectional genes indicated its potential role in the VSMCs phenotypic switching. (**D, E**) Immunostaining and normalized fluorescence intensity analysis of KLF5 in the atherosclerotic artery from human patient. KLF5 (red), α-SMA (green), DAPI (blue). Scale bar, 20 μm, n=3. (**F, G**) Immunostaining and normalized fluorescence intensity analyses of KLF5 in HASMCs subjecting to different IFSS. KLF5 (red), DAPI (blue). Scale bar, 20 μm, n=3. (**H, I**) Western blot and quantitative analysis of KLF5 in HASMCs subjecting to IFSS. GAPDH is used as loading control, n=3. (**J**) mRNA levels of *KLF5* were analyzed by qRT-PCR. The relative abundances of transcripts were quantified and normalized to *Gapdh*, n=3. (**K, L**) Expression of KLF5 in the cytoplasm and quantitative analysis subjecting to IFSS. GAPDH was used as a loading control, n=3. (**M, N**) Expression of KLF5 in the unclear and quantitative analysis under IFSS. Lamin B was used as a loading control, n=3. (**O**) The ratio of KLF5 expression in different fractions of HASMCs. Data are shown as mean ± SEM, and statistical analysis is performed by one-way analysis of variance followed by the Tukey test.

Given the involvement of the increased IFSS in VSMCs phenotypic switching and calcification, our subsequent objective was to investigate the underlying biomechanical mechanisms responsible for the induction of VSMCs phenotypic switching by increased IFSS. We performed transcriptome sequencing of VSMCs exposed to increased IFSS. In comparison to VSMCs under normal IFSS, the volcano plot exhibited 393 upregulated and 385 downregulated genes in VSMCs upon increased IFSS stimulation (**Figure 6A**). Gene ontology enrichment analysis showed that increased IFSS influences cell proliferation, metabolism, and differentiation, all of which are tightly associated with phenotypic switching 5 (**Figure S6A**). These data further support the critical role of increased IFSS in initiating VSMCs phenotypic switching.

Transcription factors (TFs) and co-transcription factors (co-TFs) directly regulate gene transcription by binding to the promoter region of the target genes, finally resulting in the expression change 43. To identify the TFs and co-TFs involved in IFSS-induced VSMC phenotypic switching, we conducted a Venn analysis by comparing the differentially expressed genes with known human TFs and co-TFs (**Figure 6B**). Notably, KLF5 exhibited the most significant increase among the differentially expressed TFs and co-TFs associated with phenotypic switching of VSMCs (**Figure 6C**), suggesting its potential role in driving phenotypic switching of VSMCs upon increased IFSS stimulation. This observation was supported by the high expression of KLF5 in the human atherosclerotic artery (**Figure 6D** and** 6E**) and the mouse atherosclerotic artery (**Figure S6B**). To further elucidate whether KLF5 responds to increased IFSS after atherosclerosis initiation, we applied 3 dyn/cm^2^ shear stress on VSMCs and examined the expression change of KLF5. Elevated expression and nuclear accumulation of KLF5 were observed in VSMCs subjected to increased IFSS (**Figure 6F** and** 6G**). Moreover, both the protein and mRNA levels of KLF5 were significantly increased (**Figure 6H-J**). It is known that the transcriptional activation of KLF5 is regulated by its nuclear shuttling and reduced expression in the cytoplasm 44. In VSMCs subjected to increased IFSS, the expression levels of cytoplasmic and nuclear KLF5 positively correlated with the magnitude of IFSS (**Figure 6K-N**), and an increased ratio of nuclear/cytoplasmic KLF5 was detected (**Figure 6O**). Taken together, these findings indicate that KLF5 is responsive to increased IFSS in the atherosclerotic artery.

We further investigated whether KLF5 regulates VSMCs phenotypic switching in response to increased IFSS. We generated a KLF5 knockdown cell line (referred to as shKLF5-HASMCs) using shRNA technology and confirmed the knockdown efficiency through western blot and qRT-PCR assay. Remarkably, KLF5 expression was significantly decreased at both the protein and mRNA levels (**Figure S6C-F**). As anticipated, inhibiting KLF5 reverses the decreased expression of contractile markers in VSMCs exposed to increased IFSS. However, the synthetic marker MMP2 did not increase in the shKLF5-HASMCs after increased IFSS exposure (**Figure S6G-J**). Immunostaining also indicated that another synthetic marker, MMP9, remained constant in shKLF5- HASMCs even when subjected to increased IFSS (**Figure S6K-M**). Intriguingly, at the gene level, the expression of contractile markers (*ACTA*2, *TAGLN*, and *CNN*1) was restored in shKLF5-HASMCs subjected to increased IFSS (**Figure S6N-P**). Conversely, deficiency in KLF5 mitigated the expression of the synthetic marker *MMP*2 in VSMCs exposed to the increased IFSS (**Figure S6Q**). In summary, our findings demonstrate that the transcription factor KLF5 regulates the phenotypic switching of VSMCs.

### The Nuclear Accumulation of KLF5 Is Dependent on the MAPK Signaling Pathway

**Figure 7:**
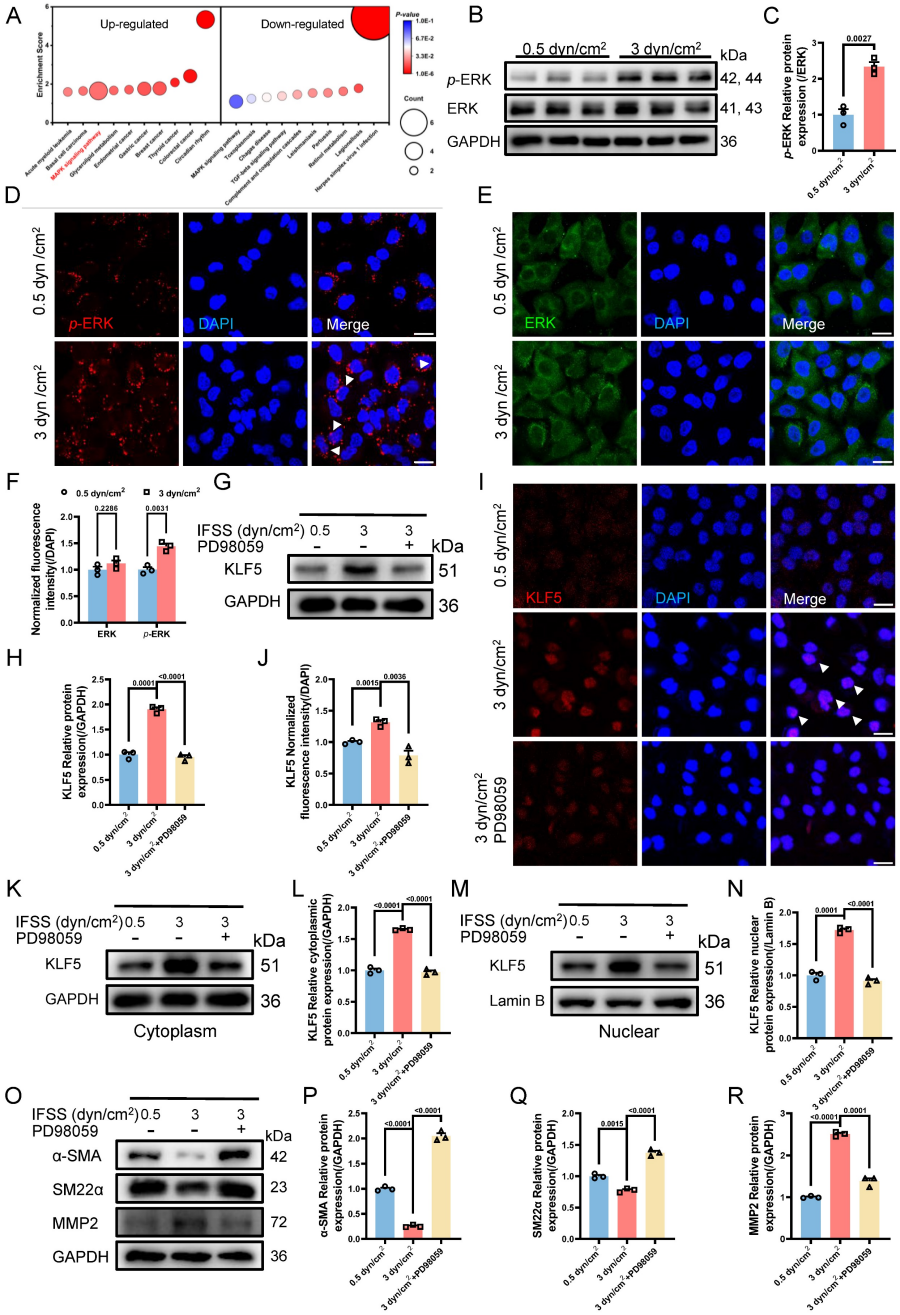
** MAPK signaling pathway initiates KLF5 to promote VSMCs phenotypic switching.** (**A**) RNA-seq analysis identified the MAPK signaling pathway as a candidate regulator of VSMCs phenotypic switching after 3 dyn/cm^2^ IFSS loading. (**B, C**) Western blot and quantitative analysis of phosphorylated ERK (*p*-ERK) and ERK in HASMCs subjecting to increased IFSS. GAPDH is used as loading control, n=3. (**D-F**) Immunostaining and normalized fluorescence intensity analyses of *p*-ERK and ERK in HASMCs. White arrows signify the nuclear localization of *p*-ERK. *p*-ERK (red), ERK (green), and nuclei were counterstained with DAPI (blue). Scale bar, 20 μm, n=3. (**G, H**) Western blot and quantitative analysis of KLF5 in HASMCs pre-treated with MAPK inhibitor (PD98059) upon IFSS stimulation. GAPDH is used as a loading control, n=3. (**I, J**) Immunostaining and normalized fluorescence intensity analysis of KLF5 in HASMCs pre-treated with MAPK inhibitor (PD98059) upon IFSS stimulation. KLF5 (red), nuclei were counterstained with DAPI (blue). Scale bar, 20 μm, n=3. (**K, L**) Expression of KLF5 in the cytoplasm and quantitative analysis pre-treated with MAPK inhibitor (PD98059) subjecting to IFSS. GAPDH was used as a loading control, n=3. (**M, N**) Expression of KLF5 in the nuclear and quantitative analysis pre-treated with PD98059 under IFSS. Lamin B was used as a loading control, n=3. (**O-R**) Western blot analysis of phenotypic switching indicators (α-SMA and SM22α, contractile marker. MMP2, synthetic marker) in HASMCs pre-treated with MAPK inhibitor (PD98059) and quantitative analysis of phenotypic switching indicators upon IFSS stimulation. GAPDH is used as a loading control, n=3. Unpaired t-tests were used to compare variables with normal distribution and homogeneity of variance. All data are presented as mean ± SEM.

To elucidate the activation of KLF5 by increased IFSS, leading to VSMCs phenotypic switching, we conducted KEGG pathway analysis based on the transcriptome sequencing results. Remarkably, the MAPK signaling pathway was found both in the top ten up-regulated and down-regulated KEGG pathway analysis, emerging as the most potent regulator of KLF5 activation (**Figure 7A**). To investigate this further, we examined the expression of the extracellular signal-regulated kinase (ERK), a downstream component of the classical MAPK signaling pathway 45, in VSMCs exposed to 3 dyn/cm^2^ IFSS. We observed a constant tendency in ERK expression accompanied by enhanced phosphorylated ERK (*p*-ERK) (**Figure 7B** and** 7C**). Immunostaining demonstrated this expression tendency as well and showed the nuclear translocation of *p*-ERK in VSMCs after 3 dyn/cm^2^ IFSS loading (**Figure 7D**-**F**, indicated by white arrows). Subsequently, PD98059 inhibitor was employed to suppress the activation of MAPK signaling pathway under increased IFSS stimulation. After exposure to PD98059, the key components of the MAPK signaling pathway (Ras, Raf, ERK and *p*-ERK) significantly declined in VSMCs even subjecting to increased IFSS (**Figure S7A-D**). Accordingly, the nuclear outward shuttling of *p*-ERK, which was inhibited by PD98059, further confirmed the repression of MAPK pathway activation in IFSS-stimulated VSMCs (**Figure S7E** and** S7F**). These data together led us to explore whether the MAPK signaling pathway could activate KLF5 to initiate VSMCs phenotypic switching. Inhibition of the MAPK signaling pathway with the PD98059 inhibitor resulted in decreased KLF5 expression in VSMCs subjected to increased IFSS (**Figure 7G** and** 7H**).

Additionally, immunostaining shows that blocking MAPK signaling pathway excludes the KLF5 from the nucleus in increased IFSS-exposed VSMCs (**Figure 7I** and** 7J**). This immunostaining results was confirmed by the western blot assay for KLF5 in the nuclear fraction of VSMCs subjected to increased IFSS (**Figure 7K-N**), indicating that KLF5 nuclear accumulation is dependent on the MAPK signaling pathway. Next, we evaluated the phenotypic switching of VSMCs after inhibiting MAPK upon IFSS stimulation. The contractile markers (α-SMA and SM22α) showed remarkably elevated expression, while the synthetic marker (MMP9) exhibited declined expression in VSMCs subjected to PD98059 exposure even with stimulation of increased IFSS (**Figure 7O-R**, **Figure S7G** and **S7H**). These results provide solid evidence that the MAPK signaling pathway is central for the IFSS-induced VSMCs phenotypic switching.

### EGFR Initiates the Increased IFSS-based Mechanical Cues Transducing to MAPK Signaling

**Figure 8:**
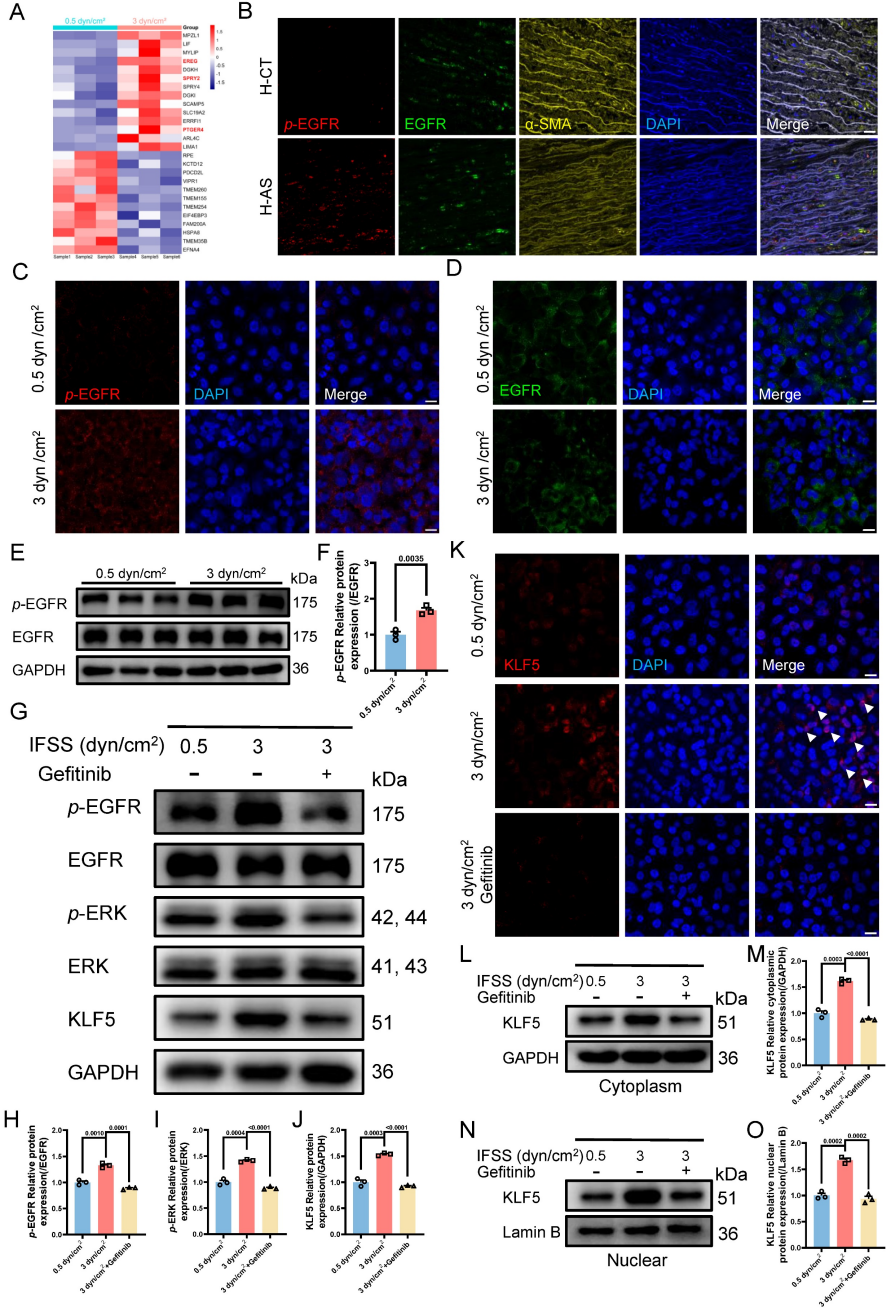
**The IFSS-based mechanical clue is initiated by EGFR.** (**A**) Heatmap illustrates the changes in membrane-bound genes after increased IFSS exposure. 14 genes show a significant increase after 3 dyn/cm^2^ IFSS loading. Three genes highlighted in red are associated with the EGFR receptor. (**B**) Immunostaining shows the location and expression of *p*-EGFR (red), EGFR (green) and VSMCs phenotype (α-SMA, yellow) in human atherosclerotic arteries. Cell nuclei were counterstained with DAPI (blue). The red channel was linearly enhanced by 20% for clear view. Scale bar, 20 μm, n=3. (**C, D**) Immunostaining of *p*-EGFR and EGFR in HASMCs. *p*-EGFR (red), EGFR (green), and nuclei were counterstained with DAPI (blue). Scale bar, 20 μm, n=3. (**E, F**) Western blot and quantitative analysis of phosphorylated EGFR (*p*-EGFR) and EGFR in HASMCs with elevated IFSS. GAPDH is used as loading control, n=3. (**G-J**) Western blot and quantitative analysis of *p*-EGFR, EGFR,* p*-ERK, ERK and KLF5 in HASMCs with pre-treatment of EGFR inhibitor (gefitinib) followed by IFSS stimulation. GAPDH is used as a loading control, n=3. (**K**) Immunostaining of KLF5 in HASMCs pre-treated with EGFR inhibitor (gefitinib) upon IFSS stimulation. KLF5 (red), nuclei were counterstained with DAPI (blue). Scale bar, 20 μm, n=3. (**L, M**) Expression of KLF5 in the cytoplasm and quantitative analysis in HASMCs pre-treated with gefitinib subjecting to IFSS. GAPDH was used as a loading control, n=3. (**N, O**) KLF5 expression in nuclear and quantitative analysis in HASMCs pre-treated with gefitinib under IFSS. Lamin B was used as a loading control, n=3. Unpaired t-tests were used to compare variables with normal distribution and homogeneity of variance. All data are presented as mean ± SEM.

Our findings underscore the critical role of the MAPK-KLF5 signaling axis in VSMC phenotype switching. It raises another interesting question: how is IFSS signaling propagated from the exterior of the VSMC cell to the nucleus interior? Based on RNA sequence data, we identified 26 differently expressed genes linked to membrane proteins and 14 genes exhibited significant upregulation after increased IFSS stimulation (**Figure 8A**). Among them, three genes—*EREG*, *SPRY2*, and *PTGER4*—were found to associate with the epidermal growth factor receptor (EGFR), which has been identified as a mechanotransduction mediator 46, 47. Subsequently, these three genes were validated by qPCR, aligning with sequencing data (**Figure S8A-C**), suggesting that EGFR is the potential candidate sensing the IFSS-based mechanical clue. After focusing on EGFR, we accordingly performed immunofluorescence staining of phosphorylated EGFR (*p*-EGFR) and EGFR using patient and mouse samples respectively (**Figure 8B** and **S8E**). The phosphorylation of EGFR refers to its activation 48. Interestingly, the elevated *p*-EGFR was observed in the AS group consisting with a reduced α-SMA expression both in the human and mouse samples while EGFR remains stable (**Figure S8D** and** S8F**). It indicates that EGFR is activated in the synthetic VSMCs. Furthermore, we conducted an immunostaining assay to determine whether EGFR is activated in synthetic VSMCs mediated by increased IFSS. Upon increased IFSS stimulating,* p*-EGFR shows a remarkably higher expression while EGFR does not show an increased expression (**Figure 8C** and** 8D**). Western blot assay was used to confirm the immunostaining data. The results show that* p*-EGFR has a remarkably higher expression while EGFR remains a stable expression, which resulting in a higher ratio of *p*-EGFR/EGFR (**Figure 8E** and** 8F**). The above-mentioned data together indicated that EGFR could response to increased IFSS.

We have demonstrated the nuclear executor function of KLF5 and biomechanical signal transducing role of MAPK signaling pathway in phenotypic switching of VSMCs mediated by increased IFSS, respectively. However, we still do not know whether EGFR activates MAPK-KLF5 signal axis. Therefore, we employed EGFR inhibitor (gefitinib) to reveal its function in the IFSS-based mechanical clue 47. Firstly, we validated the blocking proficiency of gefitinib for EGFR in VSMCs after 3 dyn/cm^2^ IFSS loading. The reduced *p*-EGFR and stable EGFR suggested that gefitinib could inhibit the activation of EGFR induced by increased IFSS (**Figure 8G-H** and **S8G-H**). We further investigated the potential activation of key MAPK signaling molecules, *p*-ERK and ERK, under increased IFSS conditions in the presence of gefitinib. Gefitinib treatment led to a notable reduction in *p*-ERK levels (**Figure 8G** and **8I**), suggesting the absence of downstream MAPK pathway activation following EGFR blockade. Moreover, total KLF5 expression was markedly attenuated following gefitinib treatment (**Figure 8G, 8J** and** 8K**). Meanwhile, immunostaining of KLF5 in VSMCs with gefitinib treatment showed obvious extranuclear transportation even under IFSS stimulation (**Figure 8L** and **8M**), which is accompanied by a substantial reduction of KLF5 in nuclear fraction (**Figure 8N** and** 8O**). These findings reinforce the pivotal role of EGFR within the mechanotransduction signaling cascade.

Subsequent investigations focused on the interplay between EGFR inhibition and VSMC phenotypic switching. As we expected, contractile markers (α-SMA and SM22α) exhibited a significant upregulation, whereas synthetic markers (MMP2 and MMP9) were downregulated in VSMCs subjected to both increased IFSS and gefitinib exposure (**Figure S8I-M**). Taken together, these results imply that IFSS-activated EGFR initiates the MAPK-KLF5 signaling axis, culminating in phenotypic switching of VSMCs.

## Discussion

VSMCs play a critical role in preserving the integrity of blood vessel walls and regulating vascular tension 49. However, their pathological behavior, characterized by enhanced motility and proliferation, is closely linked to the development of atherosclerosis. Phenotypic switching of VSMCs, where they transition from a contractile to a synthetic phenotype, has been implicated in the initiation and progression of atherosclerosis 5.

After phenotypic switching, VSMCs lose the contractile phenotype marker gene, including smooth muscle cell myosin heavy chain (MYH11), 22-kDa SMC lineage-restricted protein (SM22α/tagln), and express synthetic marker genes, such as *MMP2/9*. Upon phenotypic switching, VSMCs activate inflammatory pathways, leading to increased macrophage recruitment and the advancement of atherosclerosis 50. Therefore, understanding VSMCs phenotypic switching has been recognized as a fundamental aspect in atherosclerosis research. More importantly, investigating the regulatory factors and molecular mechanisms driving phenotypic switching is necessary for developing therapeutic strategies targeting VSMCs. However, the lack of clarity regarding the regulatory factors and underlying molecular mechanisms has hindered the development of direct interventional therapies to prevent phenotypic switching in VSMCs.

Various stimuli, such as endothelial injury, pro-inflammatory cytokines, oxidative stress, extracellular matrix (ECM), and mechanical force, have been identified as triggers for phenotypic switching in VSMCs 51, 52. Among these stimuli, biomechanical factors have gained significant attention due to their direct regulatory role and their potential contribution to major arterial diseases through mechanobiological instabilities 10, 53, 54. Specifically, IFSS plays a pivotal role in regulating various biological behaviors of VSMCs and has been associated with major arterial diseases 10, 12, 13. However, accurately determining the precise value of IFSS experienced by VSMCs in atherosclerotic arteries has been challenging. Fortunately, the rapid progress in computational fluid dynamic simulations enabled us to estimate the *in vivo* IFSS realistically 55. To address this issue, we employed computational fluid dynamic simulations and incorporated patient data to estimate IFSS values with greater accuracy. To our knowledge, this is the first time that the precise value of IFSS in atherosclerotic artery was calculated based on the parameters from human artery samples. Subsequently, we conducted *in vitro* experiments to validate the promoting role of IFSS from our numerical calculation in regulating VSMCs phenotypic switching. After subjecting to the calculated atherosclerotic IFSS, phenotypic switching of VSMCs was observed, which is consistent with the synthetic VSMCs in human atherosclerotic arteries. Our study successfully confirmed the role of increased IFSS in promoting VSMCs phenotypic switching, emphasizing its significance in the context of atherosclerosis. Therefore, increased IFSS plays a decisive role in the occurrence and progression of VSMCs phenotypic switching and holds potential as a diagnostic indicator for atherosclerosis. On the other hand, extensive research has been conducted on IFSS in many diseases, including cancer and Alzheimer's disease, utilizing it as a quantitative physical parameter for disease investigation, clinical diagnosis, and treatment 56, 57. Thus, these findings highlight the potential of IFSS as a diagnostic indicator for atherosclerosis. However, studying interstitial flow presents significant challenges, necessitating the development of innovative approaches to effectively capture and assess its variations.

In addition to phenotypic switching, the secretion of EVs derived from VSMCs significantly elevated after exposure to increased IFSS. These EVs played a role in promoting VSMCs calcification, representing another form of phenotypic switching. Although cargos of the pro-calcified EVs is not explored in this study, it has been shown that sphingolipid phosphodiesterase 3, phosphatidylserine, annexin A6, and microRNAs (e.g. miR143, miR221, and miR222) contained in EVs are the potential mediator for other cells calcification 58-60. Previous studies have shown that externally added VSMC-derived EVs induce phenotypic switching under oxidative stress 20. Therefore, we wondered whether the synthetic VSMCs secreted more EVs or whether the EVs accelerated the phenotypic switching of VSMCs in the atherosclerotic artery. Co-culturing VSMCs with EVs derived from synthetic VSMCs did not induce phenotypic switching in neighboring contractile VSMCs. Our investigation revealed that phenotypic switching with increased IFSS stimulation acts as an upstream regulator of pro-calcified EVs secretion. Besides, other research indicated that the mechanism underlying the observed correlations between mechanical stress and EV biogenesis is complex and depends significantly on both the parent cell type and the nature of the applied stress 61. Therefore, the relationship between shear stress and the release of EVs remains a focal point for future research in cardiovascular diseases 62.

The molecular mechanisms underlying increased IFSS-induced phenotypic switching of VSMCs are not fully understood. Various signaling pathways, including DNA methylation/TET methylcytosine dioxygenase 2, Hippo-YAP signaling pathway, Notch signaling pathway, and integrin β3-mediated signaling cascades, have been implicated in regulating VSMC phenotypic switching 63. However, whether these pathways response to IFSS is not known. Here, we clarified that MAPK signaling pathway is activated by increased IFSS in VSMCs through conducting RNA-sequence and pharmaceutical inhibition. Next, we sought to unravel the executor responding to the biomechanical clue passed by MAPK signaling pathway. Krüppel-like factors (KLFs) are a group of DNA-binding transcription factors involved in the development, metabolism and other cellular mechanisms 64. Unlike the well-known KLF4, the mechanobiological role of KLF5 in IFSS-induced phenotypic switching of VSMCs remains unclear 44, 65-68. Our experimental findings revealed that KLF5 mediates phenotypic switching under increased IFSS conditions. The upregulation of total and nuclear KLF5 in VSMCs exposed to increased IFSS suggests its transcriptional regulatory function on synthetic phenotype genes. As a basic transcriptional factor, KLF5 is well known to regulate target gene transcription 69. The expression and activation of KLF5 are regulated by various signaling pathways and post-translational modification. The signaling pathways that regulate KLF5 expression have mostly been explored, with evidence showing that FOXO1 and YAP promoted KLF5 expression in cardiomyocytes and renal tubular cells respectively via direct binding on the KLF5 promoter 70, 71. The phosphorylated KLF5 on the different sites (such as S153 and S303) promotes the transactivating function of KLF5. In contrast, SUMOylation on K162 and K209 by enzyme SUMO1 converts KLF5 from a transcription activator to an inhibitor. On the other hand, activation of KLF5 could initiated some signaling pathway. For example, the loss of KLF5 acetylation at K358 in basal progenitors promotes luminal commitment by activating Notch signaling 72. Additionally, KLF5 is identified as the responsive gene to activating Wnt signaling pathway 73. Regarding therapeutic potential, KLF5 inhibition has been proposed as a strategy for treating atherosclerosis. Preclinical studies showed that systemic pharmacological KLF5 inhibition protects against heart disease, although prolonged inhibition may lead to cardiomyopathy 44. Therefore, understanding the spatial and temporal expression profile of KLF5 during disease progression is necessary to identify the optimal timing and extent of an inhibitory intervention.

EGFR has been identified as a mechanosensitive molecule, when subjected to compressive forces on airway epithelial cells, initiates an EGFR-dependent mechanotransduction pathway, culminating in enhanced phosphorylation of downstream kinases 74. In ovarian cancer cells, abnormal hemodynamics can consistently elevate EGFR expression and activity, primarily through decreased receptor degradation and increased recycling, which promotes cell proliferation, survival, and chemoresistance 75, 76. These findings highlight the pivotal role of EGFR in mechanotransduction processes. Additionally, post-translational modification contributes to the activation of EGFR upon biomechanical stimuli. For example, methylation of EGFR at Arg 1175 by PRMT5 enhances its phosphorylation at Tyr 1173, and critically influences ERK activity within the EGFR signaling pathway 77. The intricate balance between methylation and phosphorylation of EGFR finely regulates various cellular processes response to mechanical stimuli. Our results suggest that IFSS stimulation significantly upregulated the expression of *p*-EGFR in VSMCs. Furthermore, after inhibiting EGFR activation in VSMCs upon increased IFSS, the downstream factor *p*-ERK was markedly decreased. Collectively, we further validated the crucial role of EGFR in converting the biomechanical stimulus into a biochemical signal that activates the downstream MAPK signaling pathway.

Through the global gene expression profile and pharmacological inhibition, we confirmed that the MAPK-ERK signaling pathway acts as the upstream regulator of KLF5 in the transduction of flow-based mechanical cues. Pharmacological inhibition of ERK impedes the increased IFSS-induced nuclear accumulation of KLF5 and suppresses its expression. These findings align with previous studies showing the activating MAPK signaling pathway in VSMCs after sphingosine 1-phosphate (S1P) stimulation, which also results in an elevated expression of KLF5^78^. Blocking MAPK-ERK signaling has been proposed as an alternative therapy for inhibiting KLF5. Emerging technologies, such as PROteolysis TArgeting Chimeras (PROTACs) have attracted great attention for finding another approach to overcome the drug resistance caused by small molecular drugs 79. The Heightman group designed representative PROTACs of ERK1 and ERK2. After 16 h post-ERK-CLIPTAC was added to the cell, the degradation of ERK1 or ERK2 was observed 80. However, the therapeutic effect of this ERK-CLIPTAC was not investigated in atherosclerotic tissue or cells.

In conclusion, our study provides insights into the mechanobiological role of EGFR-MAPK-KLF5 signaling axis in IFSS-induced phenotypic switching of VSMCs, which is implicated in the development of atherosclerosis, targeting KLF5 or its upstream regulator EGFR and MAPK signaling pathway through small molecular drugs or emerging technologies, such as PROTACs holds promise as a therapeutic approach to prevent VSMCs phenotypic switching and ameliorate atherosclerosis. These findings contribute to our understanding of the underlying mechanisms in the progression of atherosclerosis and offer potential avenues for precision medicine and personalized treatment.

The current study is constrained by the limited availability of healthy artery tissue from normal human donors, mainly stemming from the limited availability of heart transplant surgeries. Therefore, some parameters such as thickness and porosity, essential for representing various layers of a healthy artery, are lacking, potentially leading to deviations in the calculation of IFSS. Future research should focus on increasing the sample size of healthy artery tissue and ideally integrating pathological grading into the calculation process to explore the mechanistic aspects further, while recognizing that observing enhanced IFSS in the atherosclerotic artery is still plausible. Furthermore, while we successfully verified the involvement of the KLF5 gene in the cell IFSS loading model through genetic blocking, its role *in vivo* remains to be validated. Prior studies have indicated that systemic and conditional KLF5 gene deletions lead to embryonic lethality, presenting a challenge for *in vivo* experiments 81, 82. To overcome this hurdle, we propose employing adeno-associated virus (AAV) in combination with CRISPR/Cas9, which offers a promising solution due to its post-birth gene editing capabilities. Moreover, our study has effectively demonstrated that IFSS activates the transcriptional function of KLF5. Nevertheless, a deeper investigation is still warranted to understand how the activated KLF5 regulates the release of pro-calcified EVs to acquire comprehensive insights into the underlying mechanisms involved.

## Supplementary Material

Supplementary materials and methods, figures and tables.

## Figures and Tables

**Table 1:** Physiological/Pathological parameters and boundary conditions used in the numerical model.

Layers	Parameters	Normal	Atherosclerosis
Lumen	Diameter, mm	19	19
	Density ρ, g/mm^3^	1.05×10^-3^	1.05×10^-3^
	Dynamic viscosity μ, g/(mm∙s)	3.5×10^-3^	3.5×10^-3^
	Inlet flow, L/min	5	5
	Outlet pressure, mmHg	70	70

Endothelium	Thickness, μm	182.2	209.5
	Density ρ, g/mm^3^	1.05×10^-3^	1.05×10^-3^
	Dynamic viscosity μ, g/(mm∙s)	3.5×10^-3^	3.5×10^-3^
	Porosity ε	0.054	0.122

Smooth Muscle Cell	Thickness, μm	1234.6	1220.33
	Density ρ, g/mm^3^	1.00×10^-3^	1.00×10^-3^
	Dynamic viscosity μ, g/(mm∙s)	3.5×10^-3^	3.5×10^-3^
	Porosity ε	0.05	0.08
	Interstitial flow velocity, μm/s	0.1/1	0.1/1

Adventitia	Thickness, μm	162.1	248.6
	Density ρ, g/mm^3^	1.05×10^-3^	1.05×10^-3^
	Dynamic viscosity μ, g/(mm∙s)	3.5×10^-3^	3.5×10^-3^
	Porosity ε	0.01	0.01

## Abbreviations

ALP: alkaline phosphatase
ApoE: apolipoprotein E-deficient
AS: atherosclerosis
α-SMA: alpha smooth muscle actin
CD31: cluster of differentiation 31
CD63: cluster of differentiation 63
CVD: cardiovascular diseases
EGFR: epidermal growth factor receptor
ERK: extracellular signal-regulated kinases
EVs: extracellular vesicles
FOXO1: forkhead box o1
IFSS: interstitial fluid shear stress
KLF4: krüppel-like factor 4
KLF5: krüppel-like factor 5
MAPK: mitogen-activated protein kinases
MMP2: matrix metallopeptidase 2
MMP9: matrix metallopeptidase 2
MYH11: myosin heavy chain 11
*p*-ERK: phosphorylated extracellular signal-regulated kinases
PROTACs: proteolysis targeting chimera
Runx2: runt-related transcription factor 2
TF: transcription factor
TSG101: tumor susceptibility gene 101
VSMCs: vascular smooth muscle cells
WSS: wall shear stress
