# Supplementary material for: Interstitial Fluid Shear Stress Induces the Synthetic Phenotype Switching of VSMCs to Release Pro-calcified Extracellular Vesicles via EGFR-MAPK-KLF5 Pathway
Source: Int J Biol Sci. 2024 Apr 29;20(7):2727–47. doi: 10.7150/ijbs.90725 (PMC11077359; doi:10.7150/ijbs.90725)
Supplement: Supplementary file 1 — Supplementary materials and methods, figures and tables. [file ijbsv20p2727s1.pdf]

Supplemental Materials and Methods

Extracellular Vesicles Extraction

Aortic tissue samples from atherosclerotic mice (*ApoE*$^{-/-}$ mice with western diet) and cultured HASMCs subjected to IFSS were used for extracellular vesicles (EVs) extraction. After 12 weeks of a normal or high-fat diet in C56BL/6 or *ApoE*$^{-/-}$ mice, aortic tissue samples were collected and connective tissue was extracted. The samples were then washed with sterile PBS and vessels were cut into 5 mm^3 pieces. These pieces were added to DMEM high sugar medium containing 1mg/mL collagenase and incubated at 37°C for 4 h with vortex shaking every 30 min. The resulting mixture was filtered using a 40 μm filter, and the supernatant was collected and centrifuged at 500 g for 5 min at 4°C. The resulting supernatant was then further centrifuged at 10,000 g for 10 min at 4°C. Next, 0.2 mL of EVs purification solution (EX010, Shanghai, China) was added to each 1 ml of supernatant, mixed well, and left overnight at 4°C. The next day, the EVs in the tissues were obtained by centrifugation at 1500 g for 30 min at 4°C. The resulting supernatant was discarded, and the sample was centrifuged again at 1500 g for 5 min to fully remove any remaining liquid. On the other hand, the extracellular vehicles (EVs) extraction from HASMCs after subjecting to interstitial fluid shear stress (IFSS) was conducted as follows. The cell medium was collected and centrifuged at 300 g for 20 min, then at 3000 g for 20 min and finally at 10,000 g for 30 min. The resulting supernatant was aspirated and added to an ultra-separation tube. The EVs were collected by centrifugation at 100,000 g for 2 h at 4°C, after which the supernatant was discarded and the sediment at the bottom of the tube was collected as EVs. These collected EVs were used for subsequent experiments.

NanoSight Tracking Analysis

EVs deposition was resuspended in PBS to an appropriate concentration. The NanoSight Tracking Analysis (NTA) system (Particle Metrix) was used to analyze EVs. Initially, the NTA system was washed with ultrapure water, followed by the injection of 5 mL of calibration solution into the sample pool using a syringe to initiate automatic calibration. After calibration, the sample pool was washed with ultrapure water, and the test was started when the particle number was ≤ 10. EVs were appropriately diluted in PBS for further analysis.

Alizarin Reds S Staining

Arterial samples from different groups of mice were collected, and the surrounding connective tissue was removed. The target artery sections, including the aortic arch, left carotid artery, right carotid artery, descending aorta, and abdominal aorta, were fixed in 4% paraformaldehyde for 2 h and were embedded in 4 μm paraffin blocks. Subsequently, the sections were dehydrated and dewaxed using xylene and gradient ethanol. They were then immersed in Alizarin Red S staining solution (pH = 4.2) for 30 min at room

temperature. After staining, the sections were washed three or five times with water. In the case of HASMCs, the culture medium was removed, and the cells were washed three times with sterile PBS. The cells are fixed in 95% ethanol for 30 min, followed by three washes with ultrapure water at room temperature. Subsequently, the cells were stained with Alizarin Red S staining solution (pH = 4.2) in the dark for 30 min at room temperature. After removing the staining solution, the cells were washed three times with ultrapure water and then observed and photographed under a microscope.

Von Kossa Staining

Von Kossa silver staining solution was applied to the vessel sections and then irradiated with UV for 60 min. Afterward, the sections were washed with distilled water for 1 min, treated with 5% sodium thiosulfate for 2 min, and washed three times with PBS. Following the staining process, the vessel sections were dehydrated using gradient ethanol and xylene, dried, and sealed. Imaging was carried out using a film sweeper (SLIDEVIEW VS200, Olympus).

Transmission Electron Microscopy (TEM)

To validate the characteristics of EVs isolated from mice tissue or HASMCs, transmission electron microscopy (TEM) was employed. The TEM observation was performed following the previously described [1]. Images were captured by transmission electron microscopy (HT7800, Hitachi tech).

Oil Red O Stain

The aortas from C56BL/6 and *ApoE*$^{-/-}$ atherosclerotic mice were dissected, with the removal of any excess connective and adipose tissue. The aortas were then fixed in a 4% paraformaldehyde solution for 48 h. Subsequently, the aortas were sectioned from the aortic arch to the iliac artery and rinsed in water for 10 min before being soaked in 60% formaldehyde for an additional 10 min. The vessels were then stained with an Oil Red O staining solution while being agitated at 90 rpm for 10 min. After staining, the vessels were washed sequentially in formaldehyde and water for 10 min respectively and then photographed.

Wound-Healing Assay

The wound-healing assay was performed according to a previously described method[2]. Briefly, HAMSCs were cultured on sterile slides until reaching confluence. The cells were then treated with the serum-free DMEM medium or appropriate inhibitors 24 h before the experiment. Subsequently, the cells were exposed to IFSS of 0.5 dyn/cm^2 or 3 dyn/cm^2 for 8 h, respectively. A straight and uniform wound area was created with a tip of a sterile micropipette. The slides were incubated at 37°C in a 5% CO_2 environment, and photographs of the same wound area were taken at the indicated time points (0 and 24 h) using an inverted microscope (Olympus). The images were analyzed using ImageJ software, and the experiment was repeated three times

in parallel.

Immunofluorescence Staining

Artery tissue sections from different groups of mice were treated with xylene and varying concentrations of ethanol, followed by heating in a microwave for 15 min and soaking in a sodium citrate solution to repair the antigens. The sections were then maintained at room temperature. For HASMCs after IFSS loading, the cells were washed with PBS and fixed with 4% paraformaldehyde for 30 min at room temperature, followed by PBS washing. Different primary antibodies (refer to **Table S1**) were dissolved in PBS containing 1% BSA, mixed in appropriate proportions, and added uniformly to the tissue or cells overnight at 4°C. Subsequently, the secondary antibodies (including TRITC-conjugated goat anti-rabbit antibody, FITC-conjugated goat anti-mouse antibody, FITC-conjugated goat anti-rabbit antibody, ZSGB-BIO, goat anti-rabbit IgG H&L; Alexa Fluor® 594, and Alexa Fluor® 647, Abcam) were incubated for 1 h at room temperature. Nuclei staining was performed using 4'6'-diamidino-2-phenylindole (DAPI). The Tyramide SuperBoost^{TM} kit with Alexa Flour (Thermo Fisher) was employed for multi-color staining. The samples were stored in 1×PBS in the dark and observed by confocal laser scanning microscopy (CLSM, Zeiss, LSM710). The images were then post-processed using FIJI Image J software.

Quantitative Real-time Polymerase Chain Reaction Detecting System (qRT-PCR)

Total RNA was extracted from cells upon IFSS stimulation for 8 h using the Cellular Total RNA Isolation Kit (FOREGENE) following the manufacturer's instruction and was quantified through NanoDrop spectrophotometer (ThrmoFisher Scientific, Waltham, MA, USA). cDNA was synthesized from 1 μg of total RNA using the Evo M-MLV RT Mix Kit and gDNAClean for qPCR (ACCURATE BIOLOGY). qRT-PCR was performed using 2×Taq SYBRGreen® qPCR PreMix (Innovagene) and the CFX Connect Real-Time PCR detection system (Bio-Rad Laboratories Inc) to assess the expression levels of target genes. The reference gene *GAPDH* was used to normalize relative expression values, which were obtained using the delta-Ct method. The primer sequences are available in **Table S2** (Ykang Biotech Company).

Nuclear and Cytoplasmic Protein Extraction

The nuclear and cytoplasmic protein extraction was performed as described previously[1]. The nuclear and cytoplasmic extracts were prepared using the nuclear protein extraction kit (Solarbio), following the manufacturer's instructions. Lamin B was used as the internal control for nuclear protein in subsequent western blot analysis.

Western Blot

Western blot was performed according to previously described protocols[3]. Briefly, cells were lysed in cold

RIPA buffer containing 1% protease inhibitor, 1% phosphatase inhibitor (SAB) and 1% phenylmethanesulfonyl fluoride (PMSF) on slides. Proteins in the supernatant were collected after centrifugation at 12,000 g for 10 min at 4°C and quantified using the BCA kit (Beyotime Biotechnology). Each sample was then electrophoresed on 8% or 12% sodium dodecyl sulfate-polyacrylamide gel for 2-3 h and transferred to 0.22 μm Immobilon-FL polyvinylidene difluoride membranes (PVDF; Merck Millipore Ltd, Tullagreen, Carrigtwohill, Co., Cork, IRL) with TBST buffer containing 5% skimmed milk (20 mM Tris-HCl [pH 7.4], 500 mM NaCl, 0.1% Tween 20) for 2 h at room temperature. The primary antibodies listed in **Table S1** were incubated overnight at 4°C, followed by incubation with horseradish peroxidase (HRP)-conjugated anti-rabbit and anti-mouse secondary antibodies (1:3000, Solarbio) for 2 h at room temperature. Enhanced chemiluminescence (ECL, 4ABIO.) was used to visualize the bands, and the Molecular Image®ChemiDocTM XRS+ system (Bio-Rad Laboratories Inc) with Image LabTM software was utilized. Each experiment was repeated three times, and quantitative analysis was performed using FIJI Image J software.

Immunohistochemical Staining

Immunohistochemistry was conducted to detect target proteins following previously described methods[2]. Briefly, antigen repair was performed by boiling in citrate buffer (pH 6.0), and endogenous peroxidase activity was blocked with hydrogen peroxide. Aortic vascular tissue sections were stained using the appropriate primary antibody (refer to **Table S1**). Staining was visualized using the DAB method (TA-125QHPX, Thermo Scientific) according to the manufacturer's instructions.

RNA-Sequencing

Total RNA was extracted from the sample by using the TRIzol reagent (Invitrogen Company) following the manufacturer's instructions. Next-generation sequencing was performed by Aksomics, Inc[3, 4]. Quality control of total RNA was assessed using agarose gel electrophoresis, and quantification was checked using NANODROP. Agilent 2100 was used for library quality control, and library quantification was performed using qPCR. Sequencing was conducted using Illumina Hiseq 4000. After using FastQC software to detect sequence quality, the abundance of transcripts in each sample was estimated by StringTie. The FPKM value (≥0.5) of genes and transcripts were assessed using the R package Ballgown. Differentially expressed genes and transcripts were filtered based on a fold change of 1.5 or greater. Principal component analysis (PCA) was performed based on gene expression levels, and correlation analysis was conducted. Hierarchical clustering, Gene Ontology (GO), and pathway analysis were performed using genes with a fold change of 1.5 or greater.

Plasmid construction, lentiviral production, and transfection

To generate KLF5 knockdown cells, oligonucleotides were cloned into psi-LVRU6GP with the BamHI/EcoRI restriction enzyme cutting sites. The sequences of the oligonucleotides are as follows: KLF5-sense, 5'-

GGTTACCTTACAGTATCAACA-3'; KLF5-antisense, 5'-TGTTGATACTGTAAGGTAACC-3'. An empty vector was used as a control, referred to as shNT. Plasmids were transformed, propagated, and purified from DH5a E. coli host cells. To establish a HASMCs cell line with KLF5 stably silenced, HEK293-T cells were co-transfected with the viral plasmid and lentiviral packaging plasmids psPAX2 and pMD2.G. The medium after 48 h and 72 h transfection was collected, filtered through a 0.45 μm filter, and centrifuged at 4000 rpm for 30 min. The PEG precipitation was then resuspended with serum-free DMEM medium (Gibco) and used to infect HASMCs cells. Cells were screened by high glucose DMEM culture medium containing 0.5 μg/mL puromycin. After 1 week of puromycin screening, the expression levels of the KLF5 were detected using qRT-PCR and western blot.

Supplementary figures and legends

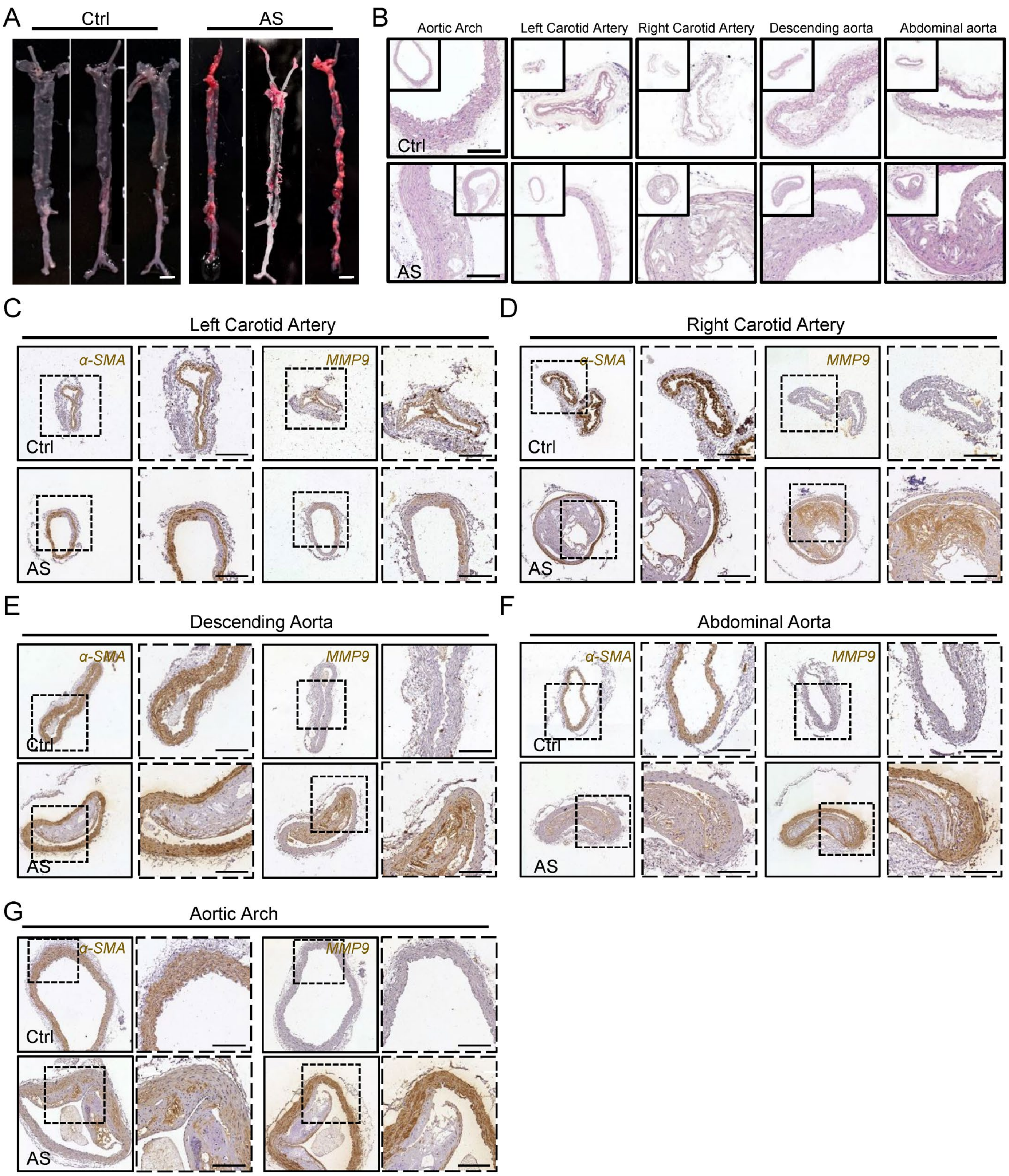

Figure S1. VSMCs undergo phenotypic switching in atherosclerotic artery of mice. (**A**) Oil Red O staining of atherosclerotic mouse aorta. Scale bar, 2 mm, n=3. (**B**) HE staining revealed the morphology of different artery segments in mouse. Scale bar, 200 μm, n=3. (**C-G**) Immunohistochemistry staining for contractile marker α-SMA and synthetic marker MMP9 in different artery segments in mouse. Scale bar, 200 μm, n=3.

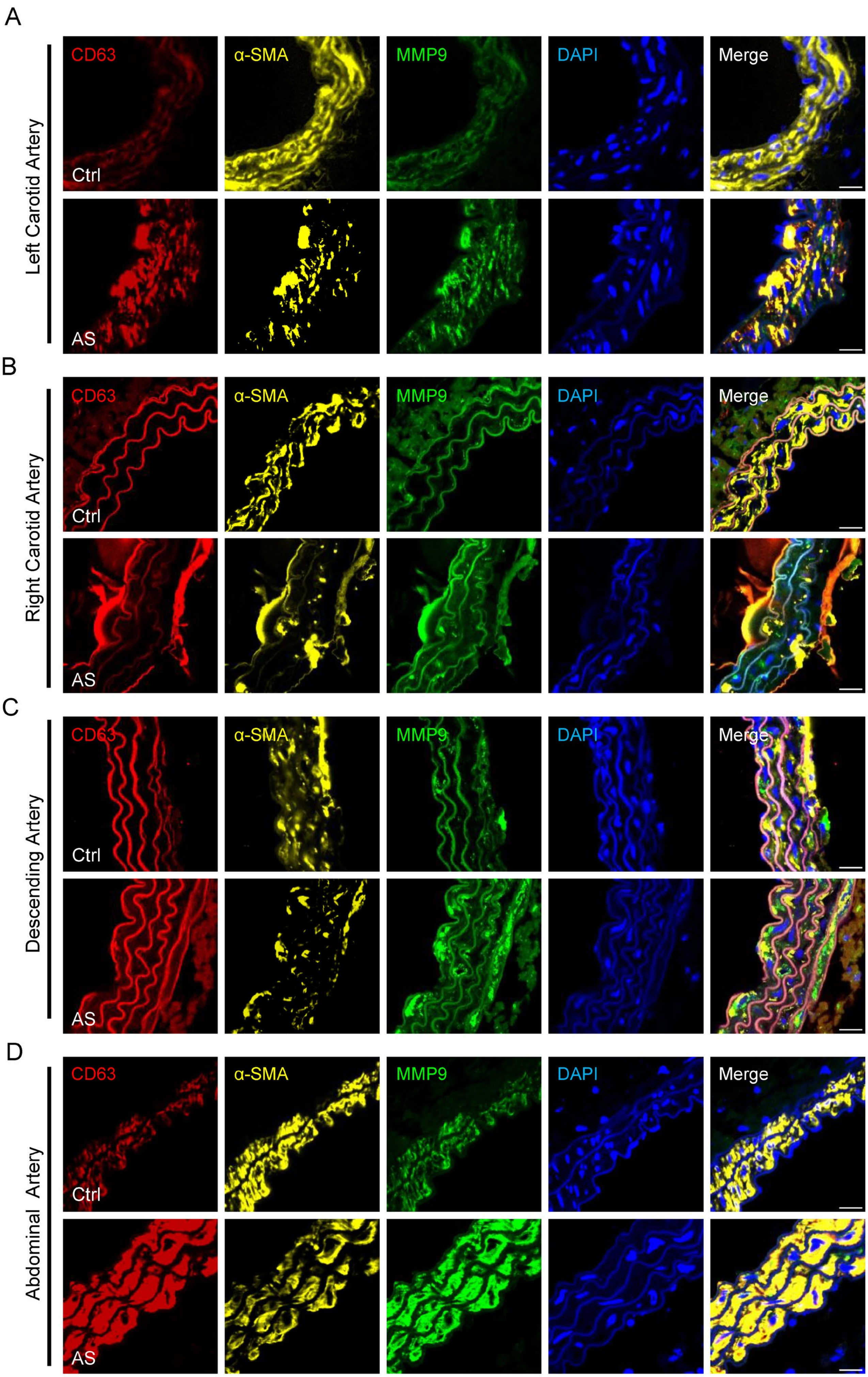
A
B
C
D
Left Carotid Artery
Right Carotid Artery
Descending Artery
Abdominal Artery
CD63
α-SMA
MMP9
DAPI
Merge
Ctrl
AS

142

Figure S2. Phenotypic switching of VSMCs is associated with increased release of EVs. (**A-D**) Immunostaining for contractile markers α-SMA, synthetic marker MMP9 and EVs marker CD63 in different artery segments of the atherosclerotic mouse. α-SMA (yellow), MMP9 (green), CD63 (red), nuclei were counterstained with DAPI (blue). Scale bar, 20 μm, n=3.

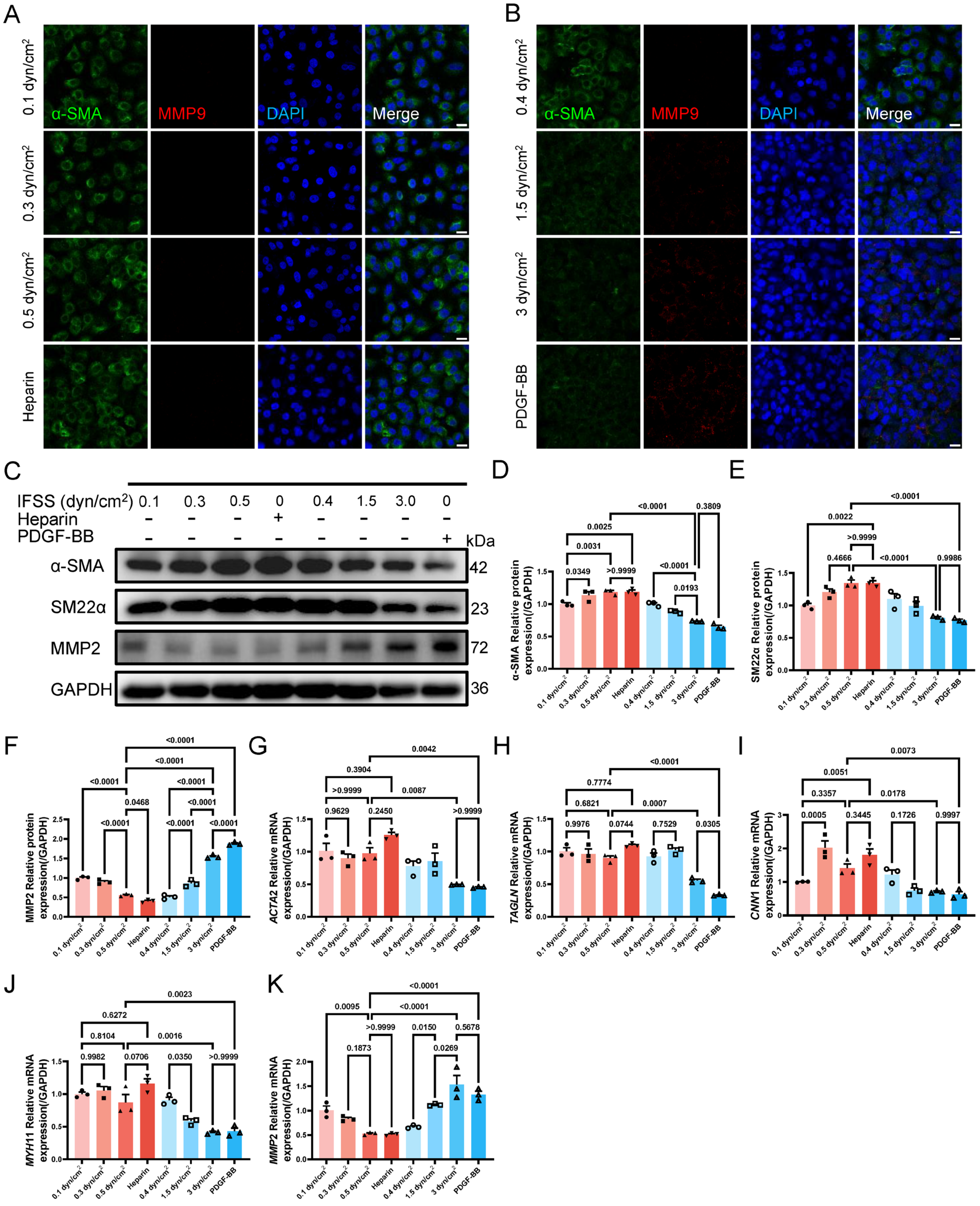

Figure S3. The screening of IFSS magnitude and *in vitro* verification. (**A**) Immunostaining demonstrated alterations in the expression of the contractile marker α-SMA (red) and the synthetic marker MMP9 (green) within HASMCs under physiological IFSS stimulation *in vitro*. The contractile HASMCs maintained by heparin treatment were set up as the negative control. Scale bar, 20 μm, n=3. (**B)** Immunostaining reveals

changes in the expression of α-SMA (red) and MMP9 (green) in HASMCs upon pathological IFSS stimulation *in vitro*. The synthetic HASMCs induced by PDGF-BB were used as the positive control. The nucleus is counterstained with DAPI (blue). Scale bar, 20 μm, n=3. (**C-F**) Western blot and quantitative analysis of HASMC phenotypic switching markers (contractile marker α-SMA, SM22α and synthetic marker MMP2) following exposure to various levels IFSS. GAPDH served as a loading control, n=3. (**G-K**) After different magnitudes of IFSS stimulating, mRNA levels of contractile markers (*ACTA*2, *TAGLN*, *CNN*1, and *MYH*11) and synthetic marker (*MMP*2) in HASMCs were examined by qRT-PCR. Transcript abundances were quantified and normalized to *Gapdh*, n=3. Data are shown as mean ± SEM, and statistical analysis was performed by one-way analysis of variance followed by the Tukey test.

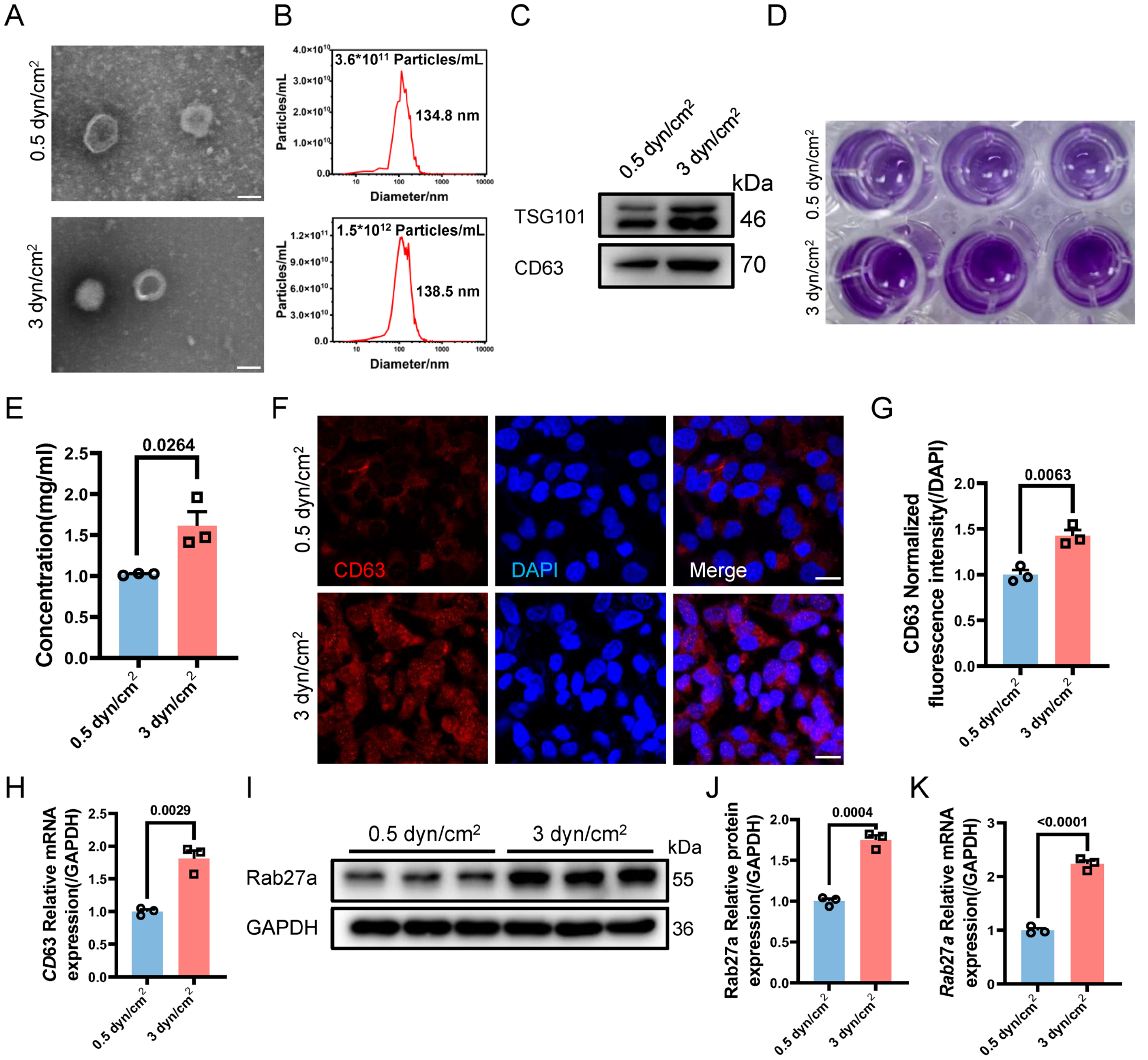

Figure S4. IFSS promotes EVs release of VSMCs. (**A, B**) TEM and NTA analysis were used to identify the HASMCs-derived EVs and quantify their abundance change respectively under increased IFSS. Scale bar, 100 μm. (**C**) Western blot analysis of markers in EVs isolated from HASMCs under increased IFSS. (**D, E**) BCA analysis was used to detect the protein concentration contained in the EVs derived from HASMCs under different IFSS stimulation, n=3. (**F-G**) Immunostaining and normalized fluorescence intensity analysis for the EVs marker protein CD63 expression HASMCs subjected to increased IFSS, CD63, red. DAPI was used to stain nuclei (blue). Scale bar, 20 μm, n=3. (**H**) qRT-PCR analysis shows the expression change of EVs marker protein CD63 in HASMCs subjected to increased IFSS, n=3. (**I, J**) Immunoblotting and quantitative analyses of Rab27a, n=3. (**K**) Gene expression levels of *Rab27a* were examined by qRT-PCR, n=3. Unpaired t-tests were used to compare variables with normal distribution and homogeneity of variance. All data are presented

as mean ± SEM.

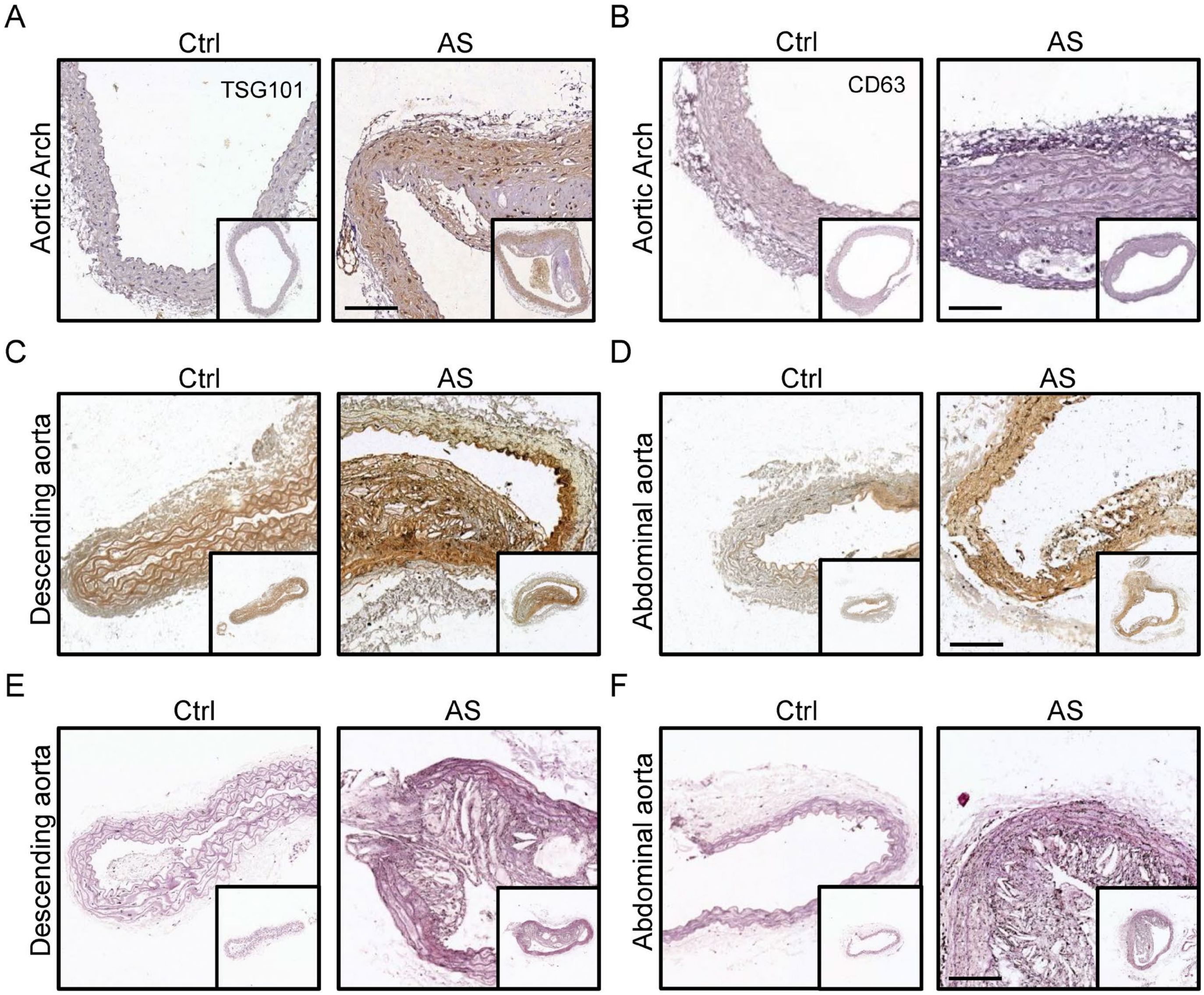

Figure S5. Increased EVs are positively linked to artery calcification. (**A, B**) Immunohistochemical staining for EVs markers TSG101 and CD63 in atherosclerotic aortic arch. (**C, D**) Von Kossa staining revealed the calcium deposition in the mouse descending aorta and abdominal aorta. Scale bar, 200 µm. (**E, F**) Alizarin red S staining revealed calcium deposition in the mouse descending aorta and abdominal aorta. Scale bar, 200 µm.

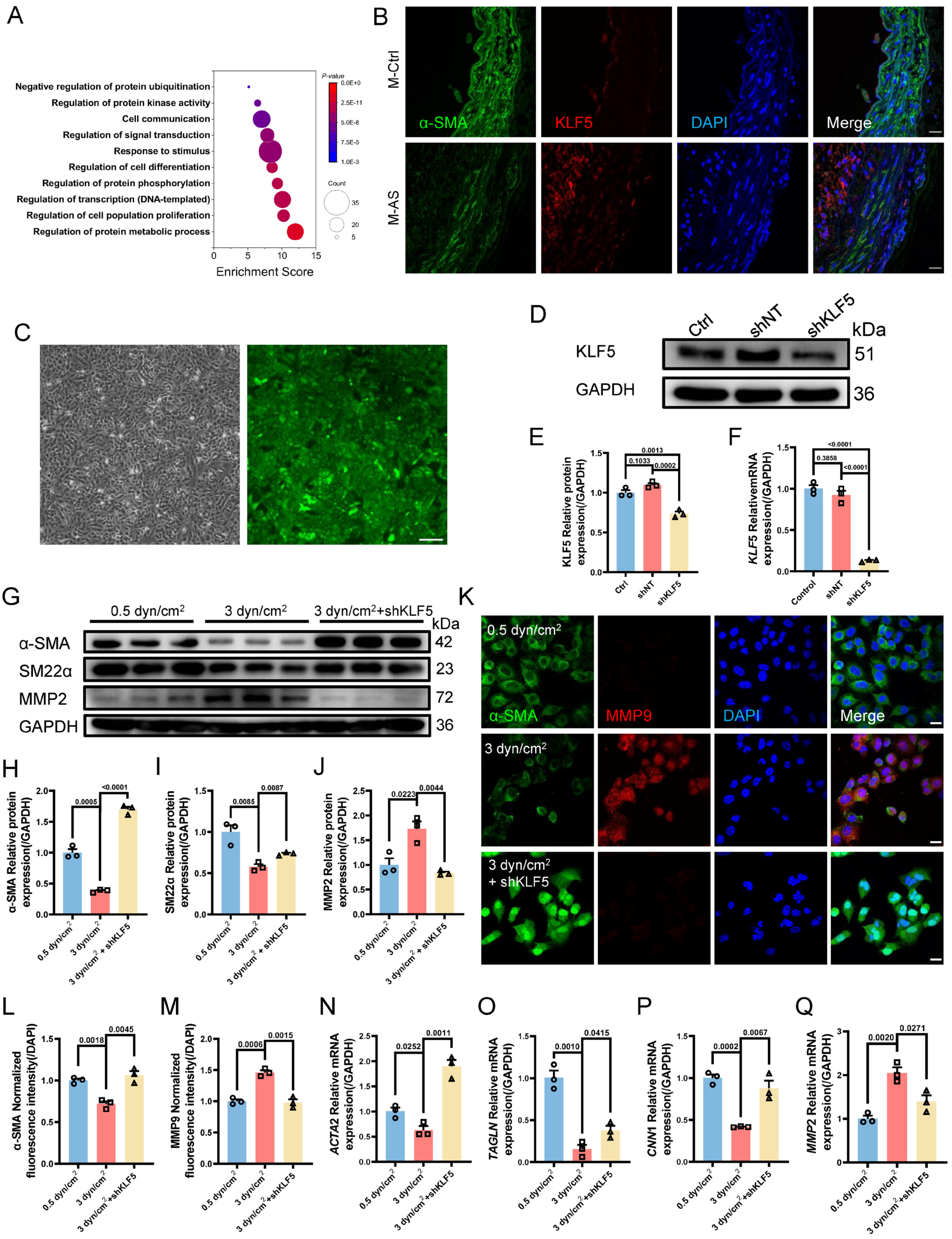

Figure S6. Deficiency of KLF5 suppresses IFSS-induced phenotypic switching of VSMCs. (A) Dot plots illustrated gene ontology (GO) enrichment terms of differentially expressed genes (DEGs) in HASMCs after

subjecting to IFSS. (**B**) Immunostaining for KLF5 in the atherosclerotic artery from mouse. KLF5 (red), α-SMA (green), DAPI (blue). Scale bar, 20 μm, n=3. (**C**) Transfected efficiency of shKFL5 RNA in HASMCs. (**D-F**) Western blot and qRT-PCR were used to confirm the deficiency of KLF5 in HASMCs transfected with shKFL5 RNA. (**G-J**) Western blot and quantitative analysis of phenotypic switching markers in shKFL5-HASMCs upon IFSS stimulation. (**K-M**) Immunofluorescence staining and normalized fluorescence intensity analyses for MMP-9 and SM22α in shKFL5-HASMCs upon IFSS stimulation. α-SMA (green), MMP9 (red), and nuclei were counterstained with DAPI (blue). Scale bar, 20 μm, n=3. (**N-Q**) mRNA levels of *ACTA*2, *TAGLN*, *CNN*1 and *MMP*2 in shKFL5-HASMCs upon IFSS stimulation. Relative abundances of transcripts were quantified and normalized to *Gapdh*, n=3. Unpaired t-tests were used to compare variables with normal distribution and homogeneity of variance. All data are presented as mean ± SEM.

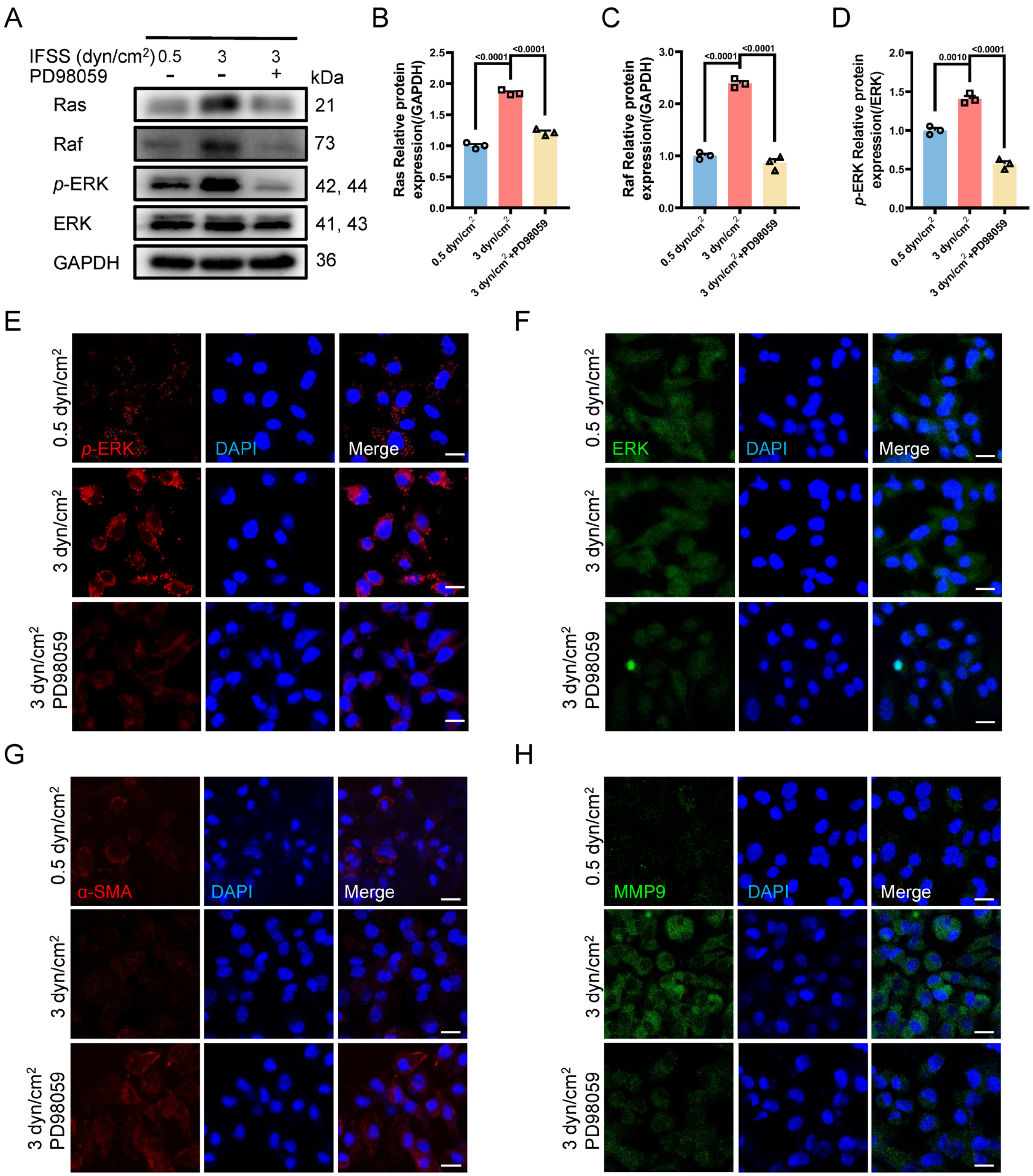

Figure S7. MAPK signal pathway contributes to IFSS-induced VSMCs phenotypic switching. (A-D) Western blot analysis revealed the activation of MAPK pathway in HASMCs with or without PD98059 treatment and quantitative analysis of key factors containing MAPK pathway upon the IFSS stimulation. GAPDH is used as a loading control, n=3. **(E, F)** Immunofluorescence staining for ERK and *p*-ERK in HASMCs pre-treated with PD98058 upon IFSS stimulation. ERK (green), *p*-ERK (red), and nuclei was counterstained with DAPI (blue). Scale bar, 20 μm. **(G, H)** Immunofluorescence staining for contractile

phenotype marker α-SMA and synthetic phenotype marker MMP9 in HASMCs pre-treated with PD98058 upon IFSS stimulation. α-SMA (red), MMP9 (green), and nuclei were counterstained with DAPI (blue). Scale bar, 20 μm. Unpaired t-tests were used to compare variables with normal distribution and homogeneity of variance. All data are presented as mean ± SEM.

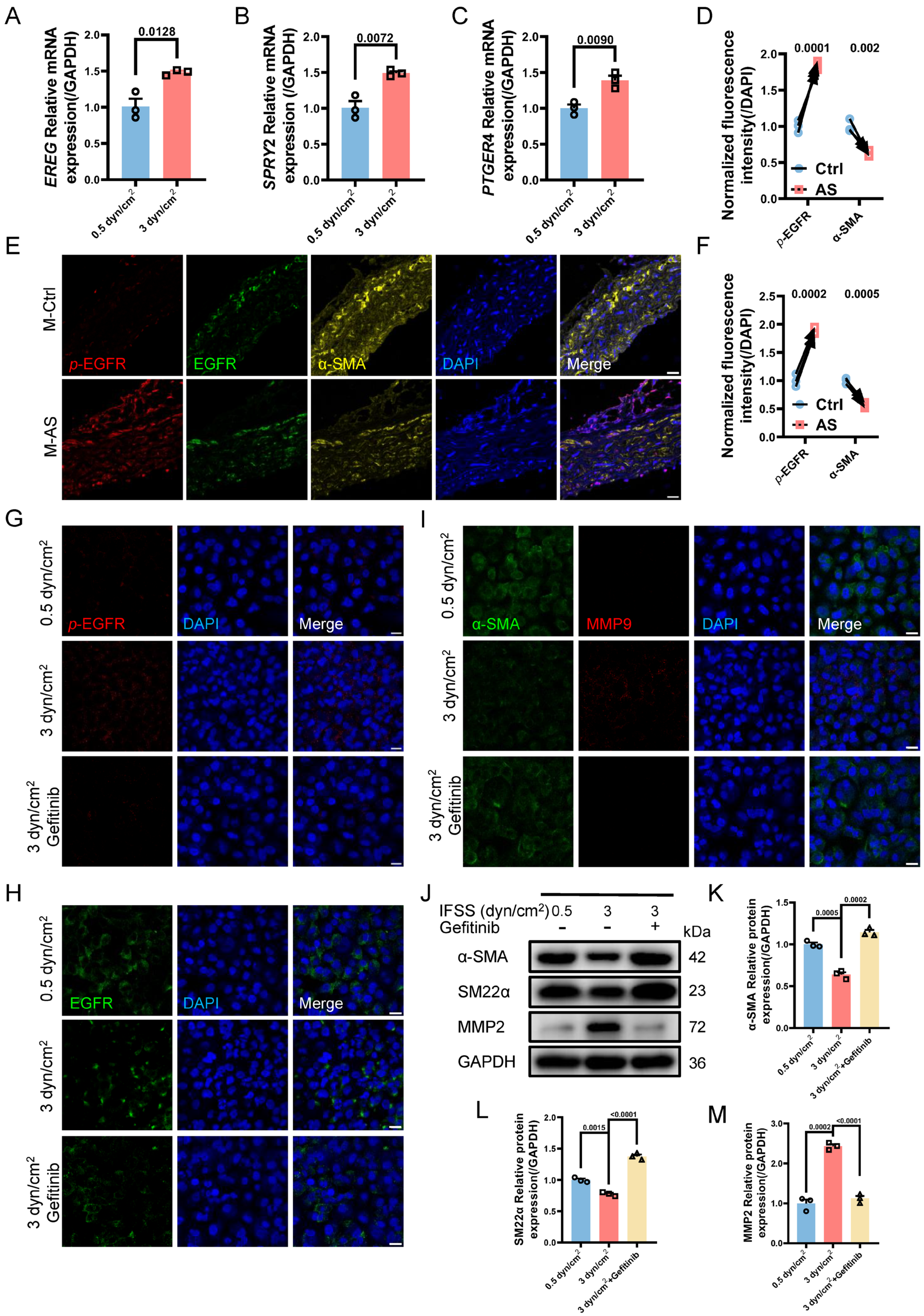
A
EREG Relative mRNA expression(/GAPDH)
0.0128
0.5 dyn/cm2
3 dyn/cm2
B
SPRY2 Relative mRNA expression (/GAPDH)
0.0072
C
PTGER4 Relative mRNA expression(/GAPDH)
0.0090
D
Normalized fluorescence intensity(/DAPI)
0.0001
0.002
Ctrl
AS
p-EGFR
α-SMA
E
M-Ctrl
M-AS
p-EGFR
EGFR
α-SMA
DAPI
Merge
F
0.0002
0.0005
G
0.5 dyn/cm2
3 dyn/cm2
3 dyn/cm2 Gefitinib
H
I
MMP9
J
IFSS (dyn/cm2) 0.5 3 3
Gefitinib - - +
kDa
SM22α
MMP2
GAPDH
42
23
72
36
K
α-SMA Relative protein expression(/GAPDH)
0.0005
0.0002
3 dyn/cm2+Gefitinib
L
SM22α Relative protein expression(/GAPDH)
0.0015
<0.0001
M
MMP2 Relative protein expression(/GAPDH)
0.0002
<0.0001

Figure S8. EGFR senses IFSS and contributes to VSMCs phenotypic switching. (**A-C**) mRNA levels of *EREG*, *SPRY*2 and *PTGER*4 in HASMCs upon increased IFSS stimulation. Relative abundances of transcripts were quantified and normalized to *Gapdh*, n=3. (**D**) Correlation analysis of *p*-EGFR and VSMCs phenotypic switching markers. (**E, F**) The expression and correlation analysis of *p*-EGFR and VSMCs phenotypic switching markers in the mouse atherosclerotic artery (*p*-EGFR, red, EGFR, green, α-SMA, yellow). Nuclei were counterstained with DAPI (blue). The red channel was linearly enhanced by 20%. Scale bar, 20 μm, n=3. (**G, H**) Immunofluorescence staining for *p*-EGFR and EGFR in HASMCs pre-treated with gefitinib upon IFSS stimulation. *p*-EGFR (red), *p*-EGFR (green), and cell nuclei was counterstained with DAPI (blue). Scale bar, 20 μm. (**I**) Immunofluorescence staining for contractile phenotype marker α-SMA and synthetic phenotype marker MMP9 in HASMCs pre-treated with gefitinib upon IFSS stimulation. α-SMA (red), MMP9 (green), and nuclei were counterstained with DAPI (blue). Scale bar, 20 μm. (**J-M**) Western blot analysis of phenotypic switching indicators (α-SMA and SM22α, contractile marker. MMP2, synthetic marker) in HASMCs pre-treated with gefitinib and quantitative analysis of phenotypic switching indicators upon IFSS stimulation. GAPDH is used as a loading control, n=3. Unpaired t-tests were used to compare variables with normal distribution and homogeneity of variance. All data are presented as mean ± SEM.

Supplementary material

Supplementary Table 1. Antibodies used in this study

Target antigen	Vendor or Source	Catalog #	Working conc	Persistent ID / URL
anti-α-SMA	Abcam	ab124964	WB: 1:1000, IHC&IF: 1:500	Recombinant Anti-alpha smooth muscle Actin antibody [EPR5368] (ab124964)
anti-MMP9	Abcam	ab283575	IF: 1:50	Recombinant Anti-MMP9 antibody [RM1020] (ab283575)
anti-SM22α	Abcam	ab14106	WB: 1:1000	Anti-TAGLN/Transgelin antibody (ab14106)
anti-MMP2	Abcam	ab92536	WB: 1:2000, IHC: 1:500	Recombinant Anti-MMP2 antibody [EPR1184] (ab92536)
anti-KLF5	Abcam	ab137676	WB: 1:2000, IF: 1:200	Anti-KLF5 antibody (ab137676)
anti-CD63	Abcam	ab134045	WB: 1:1000, IHC&IF: 1:500	Recombinant Anti-CD63 antibody [EPR5702] - Late Endosome Marker (ab134045)
Goat Anti-Rabbit IgG H&L (TRITC)	Abcam	ab6718	IF: 1:1000	Goat Anti-Rabbit IgG H&L (TRITC) (ab6718)
Goat Anti-Rabbit IgG H&L (Alexa Fluor® 488)	Abcam	ab150077	IF: 1:1000	Goat Anti-Rabbit IgG H&L (Alexa Fluor® 488) (ab150077)
Goat Anti-Mouse IgG H&L (Alexa Fluor® 488)	Abcam	ab150113	IF: 1:1000	Goat Anti-Mouse IgG H&L (Alexa Fluor® 488) (ab150113)
Goat Anti-Rabbit IgG H&L (Alexa Fluor® 647)	Abcam	ab150079	IF: 1:1000	Goat Anti-Rabbit IgG H&L (Alexa Fluor® 647) (ab150079)
Goat Anti-	Abcam	ab150080	IF: 1:1000	Goat Anti-Rabbit IgG H&L (Alexa

Rabbit IgG H&L (Alexa Fluor® 594)				Fluor® 594) (ab150080)
anti-Rab27a	Cell Signaling	69295	WB: 1:1000	Rab27A (D7Z9Q) Rabbit mAb #69295
anti-ERK	Cell Signaling	4695	WB: 1:2000	p44/42 MAPK (Erk1/2) (137F5) Rabbit mAb #4695
anti-*p*-ERK	Cell Signaling	4370	WB: 1:1000	Phospho-p44/42 MAPK (Erk1/2) (Thr202/Tyr204) (D13.14.4E) XP® Rabbit mAb #4370
anti-Ras	Cell Signaling	67648	WB: 1:1000	Ras (E8N8L) XP® Rabbit mAb #67648
anti-Raf	Cell Signaling	9422	WB: 1:1000	c-Raf Antibody #9422
anti-Lamin B1	Cell Signaling	13435	WB: 1:1000	Lamin B1 (D9V6H) Rabbit mAb #13435
anti-*p*-EGFR	Cell Signaling	3777	WB: 1:1000<br>IF: 1:500	Phospho-EGF Receptor (Tyr1068) (D7A5) XP® Rabbit mAb #3777
anti-TSG101	HUABIO	ET1701-59	WB: 1:2000,<br>IHC: 1:50	TSG101 recombinant rabbit monoclonal antibody [JJ0900] (ET1701-59)
anti-EGFR	HUABIO	ET1603-37	WB: 1:1000<br>IF: 1:200	Anti-EGFR Recombinant Rabbit Monoclonal Antibody [SZ40-19]
anti-ALP2	HUABIO	ET1601-21	WB: 1:1000	ALP Recombinant Rabbit Monoclonal Antibody [SA40-00]
anti-Runx2	SAB	29595	WB: 1:1000	RUNX2 Rabbit Polyclonal Antibody #29595
anti-CD31	SAB	52064	WB: 1:1000	CD31 Rabbit mAb #52064
anti-GAPDH	SAB	21612	WB: 1:5000	GAPDH Antibody #21612
DAPI	Solarbio	C0065	IHC&IF: 1:900	DAPI Solution, 10ug/ml, (ready-to-use)

Supplementary Table 2. Real-time PCR primer sequences used in this study

GENE	PRIMER	SEQUENCE OR CATALOGUE NUMBER	COMPANY
ACTA2	α-SMA	Forward: AGGTAACGAGTCAGAGCTTTGGC	Ykang Biotech Company
		Reverse: CTCTCTGTCCACCTTCCAGCAG	
TAGLN	SM22α	Forward: TGAAATTCATGGCTATGGAA	Ykang Biotech Company
		Reverse: TGAAACGAGTCAGCTGGATG	
CNN1	Calponin	Forward: CTGTCAGCCGAGGTTAAGAAC	Ykang Biotech Company
		Reverse: GAGGCCGTCCATGAAGTTGTT	
MYH11	Myosin heavy chain 11	Forward: CTTCCTACGGGGAATCTGTGT	Ykang Biotech Company
		Reverse: CAATGGCGTTTTGGGTGTTC	
MMP2	Matrix metallopeptidase 2	Forward: GATACCCCTTTGACGGTAAGGA	Ykang Biotech Company
		Reverse: CCTTCTCCCAAGGTCCATAGC	
VIM	Vimentin	Forward: AGTCCACTGAGTACCGGAGAC	Ykang Biotech Company
		Reverse: CATTTCACGCATCTGGCGTTC	
Rab27a	Rab27a	Forward: GCTCTGCTTGCTTTTCATTT	Ykang Biotech Company
		Reverse: TTGTCTGCTTCTTGCTGGT	
CD63	CD63	Forward: TGACTTCTGTCCTTTGCTCCT	Ykang Biotech Company
		Reverse: CCCATTATTCCCTGCTTACCT	
KLF5	Kruppel-like transcription factor 5	Forward: TTCCACAACAGGCCACTTACT	Ykang Biotech Company
		Reverse: GAGCATCTCTGCTTGTCTATCTG	
EREG	Epiregulin	Forward: GTGATTCCATCATGTATCCCAGG	Ykang Biotech Company
		Reverse: GCCATTCATGTCAGAGCTACACT	
SPRY2	Sprouty homolog 2	Forward: CCTACTGTCGTCCCAAGACCT	Ykang Biotech Company
		Reverse: GGGGCTCGTGCAGAAGAAT	
PTGER4	Prostaglandin E Receptor 4	Forward: CCGGCGGTGATGTTCATCTT	Ykang Biotech Company
		Reverse: CCCACATACCAGCGTGTAGAA	
GAPDH	GAPDH	Forward: GCTCTCTGCTCCTCCTGTTC	Ykang Biotech Company
		Reverse: ACGACCAAATCCGTTGACTC	

Supplementary Table 3. Cultured Cells

**Species**	**Vendor or Source**	**Catalog #**	**Sex**	**Persistent ID / URL**
Human Aortic Smooth Muscle Cells	Isolated from the human aorta	6110	unknown	Human Aortic Smooth Muscle Cells

Supplementary Table 4. Kits used in this study

**Description**	**Source / Repository**	**Persistent ID / URL**
Tyramide SuperBoostTM kit with Alexa Flour 488	B40922 (Thermo Fisher)	Alexa Fluor™ 488 Tyramide SuperBoost™ Kit, goat anti-rabbit IgG
Cellular Total RNA Isolation Kit	RE-03111 (FOREGENE)	Total RNA Isolation Kit (For Cells) - Foregene
Evo M-MLV RT Mix Kit and gDNAClean for qPCR	AG11728 (ACCURATE BIOLOGY)	Evo M-MLV RT Mix Kit and gDNAClean for qPCR
SYBR Green Premix Pro Taq HS qPCR Kit （Rox Plus）	AG11720 (ACCURATE BIOLOGY)	SYBR Green Premix Pro Taq HS qPCR Kit （Rox Plus）
Nuclear protein extraction kit	SN0020 (Solarbio)	Nuclear Extraction Kit
BCA kit	P0012 (Beyotime)	BCA kit

References

1. Yu H, Hou Z, Xiang M, Yang F, Ma J, Yang L, et al. Arsenic trioxide activates yes-associated protein by lysophosphatidic acid metabolism to selectively induce apoptosis of vascular smooth muscle cells. Biochim Biophys Acta Mol Cell Res. 2022; 1869: 119211.
2. Yu H, He J, Su G, Wang Y, Fang F, Yang W, et al. Fluid shear stress activates YAP to promote epithelial-mesenchymal transition in hepatocellular carcinoma. Mol Oncol. 2021; 15: 3164-83.
3. Yu H, Hou Z, Chen N, Luo R, Yang L, Miao M, et al. Yes-associated protein contributes to magnesium alloy-derivedinflammation in endothelial cells. Regen Biomater. 2022; 9: rbac002.
4. Hou Z, Xiang M, Chen N, Cai X, Zhang B, Luo R, et al. The biological responses and mechanisms of endothelial cells to magnesium alloy. Regen Biomater. 2021; 8: rbab017.
